# Human amnion mesenchymal stem cells promote endometrial repair via paracrine, preferentially than transdifferentiation

**DOI:** 10.1186/s12964-024-01656-0

**Published:** 2024-05-31

**Authors:** Xiyue Huang, Xiao Yang, Jinglin Huang, Ling Wei, Yanhua Mao, Changjiang Li, Yingfeng Zhang, Qiuhong Chen, Shasha Wu, Lele Xie, Congcong Sun, Wenwen Zhang, Jia Wang

**Affiliations:** Department of Obstetrics and Gynecology, The University-Town Hospital of Chongqing Medical University, No. 55, Daxuecheng Middle Road, Chongqing, 401331 China

**Keywords:** Intrauterine adhesion, Human amniotic mesenchymal stem cells, Paracrine, transdifferentiation, Endometrial fibrosis

## Abstract

**Background:**

Intrauterine adhesion (IUA) is one of the most severe causes of infertility in women of childbearing age with injured endometrium secondary to uterine performance. Stem cell therapy is effective in treating damaged endometrium. The current reports mainly focus on the therapeutic effects of stem cells through paracrine or transdifferentiation, respectively. This study investigates whether paracrine or transdifferentiation occurs preferentially in treating IUA.

**Methods:**

Human amniotic mesenchymal stem cells (hAMSCs) and transformed human endometrial stromal cells (THESCs) induced by transforming growth factor beta (TGF-β1) were co-cultured in vitro. The mRNA and protein expression levels of Fibronectin (FN), Collagen I, Cytokeratin19 (CK19), E-cadherin (E-cad) and Vimentin were detected by Quantitative real-time polymerase chain reaction (qPCR), Western blotting (WB) and Immunohistochemical staining (IHC). The Sprague-Dawley (SD) rats were used to establish the IUA model. hAMSCs, hAMSCs-conditional medium (hAMSCs-CM), and GFP-labeled hAMSCs were injected into intrauterine, respectively. The fibrotic area of the endometrium was evaluated by Masson staining. The number of endometrium glands was detected by hematoxylin and eosin (H&E). GFP-labeled hAMSCs were traced by immunofluorescence (IF). hAMSCs, combined with PPCNg (hAMSCs/PPCNg), were injected into the vagina, which was compared with intrauterine injection.

**Results:**

qPCR and WB revealed that FN and Collagen I levels in IUA-THESCs decreased significantly after co-culturing with hAMSCs. Moreover, CK19, E-cad, and Vimentin expressions in hAMSCs showed no significant difference after co-culture for 2 days. 6 days after co-culture, CK19, E-cad and Vimentin expressions in hAMSCs were significantly changed. Histological assays showed increased endometrial glands and a remarkable decrease in the fibrotic area in the hAMSCs and hAMSCs-CM groups. However, these changes were not statistically different between the two groups. In vivo, fluorescence imaging revealed that GFP-hAMSCs were localized in the endometrial stroma and gradually underwent apoptosis. The effect of hAMSCs by vaginal injection was comparable to that by intrauterine injection assessed by H&E staining, MASSON staining and IHC.

**Conclusions:**

Our data demonstrated that hAMSCs promoted endometrial repair via paracrine, preferentially than transdifferentiation.

**Supplementary Information:**

The online version contains supplementary material available at 10.1186/s12964-024-01656-0.

## Abstract (plain language summary)

IUA is the crucial cause of infertility in women of childbearing age, and no satisfactory treatment measures have been found in the clinic. hAMSCs can effectively treat intrauterine adhesions through paracrine and transdifferentiation mechanisms. This study confirmed in vitro and in vivo that amniotic mesenchymal stem cells preferentially inhibited endometrial fibrosis and promoted epithelial repair through paracrine, thus effectively treating intrauterine adhesions. The level of fibrosis marker proteins in IUA-THESCs decreased significantly after co-culturing with hAMSCs for 2 days in vitro. However, the level of epithelial marker proteins in hAMSCs increased significantly, requiring at least 6 days of co-culture. hAMSCs-CM had the same efficacy as hAMSCs in inhibiting fibrosis and promoting endometrial repair in IUA rats, supporting the idea that hAMSCs promoted endometrial remodeling through paracrine in vivo. In addition, GFP-labeled hAMSCs continuously colonized the endometrial stroma instead of the epithelium and gradually underwent apoptosis. These findings prove that hAMSCs ameliorate endometrial fibrosis of IUA via paracrine, preferentially than transdifferentiation, providing the latest insights into the precision treatment of IUA with hAMSCs and a theoretical basis for promoting the “cell-free therapy” of MSCs.

**Supplementary Information:**

The online version contains supplementary material available at 10.1186/s12964-024-01656-0.

## Introduction

Intrauterine adhesion (IUA), caused by intrauterine performance and infection, always lead to secondary infertility and recurrent abortion. It was characterized by loss of epithelial cells and endometrial fibrosis [1–3]. As the principal therapy, hysteroscopic transcervical resection of adhesion (TCRA) could only restore the shape of the uterine cavity [4], which was useless for endometrial fibrosis and epithelial restoration. Even with multiple adjuvant treatments such as intrauterine device (IUD) placement, uterine support balloon [5], amniotic membrane transplantation [6], and estrogen [7] to repair the damaged endometrium, the results were still unsatisfactory. Therefore, it is the focus of IUA to find effective treatments and improve fertility.

Due to their multidirectional differentiation and self-renewal ability, mesenchymal stem cells (MSCs) have been widely used to treat many diseases. The underlying mechanisms of it mainly focused on transdifferentiation [8–11] and paracrine [12–14]. Qing et al. [13] found that the damaged endometrium could be significantly repaired by transplanting human umbilical cord blood-derived MSCs (hUCB-MSCs). Moreover, the cell differentiation trajectory of scRNA-seq demonstrated that the transplanted hUCB-MSCs transdifferentiated into endometrial cells. Yu et al. [12] reported that an endometrium-conditioned medium promoted the differentiation of hAMSCs into epithelial cells in vitro, and Pkh26-labeled hAMSCs were mainly distributed in the endometrial epithelium after transplantation in vivo, which confirmed that hAMSCs could transdifferentiate into epithelial cells.

However, more studies have verified that paracrine is critical in promoting regeneration. Li et al. [15] proved the paracrine effect on endometrial stromal cells in vitro by co-culturing human amniotic epithelial cells (hAECs) with human endometrial mesenchymal stem cells (THESCs) damaged by H_2_O_2_. A recent study demonstrated that stem cells introduced to the site of injury act primarily via paracrine, activating the PI3K/AKT or FAK/ERK1/2 signaling cascades to accelerate wound contraction and healing rather than direct replacement of damaged cells [16].

Whether the differentiation ability of MSCs represented that their biological effects were achieved via transdifferentiation and whether the effect time of paracrine was earlier than that of transdifferentiation remained to be investigated. Therefore, this study aimed to reproduce both paracrine and transdifferentiation of hAMSCs in vitro in the same experimental system and to compare which was preferential.

## Methods

### Cell co-culture

IUA cell model (IUA-THESCs) (Zhejiang Meisen Cell Technology Co, Ltd) was established by induction with 10 ng/mL TGF-β1 for 48 h. Only the 3rd-6th generation of hAMSCs was used in the experiment. IUA-THESCs and hAMSCs were co-cultured in a transwell system to extract protein and RNA for detection. THESCs’ and hAMSCs’ isolated groups were used as control groups. All cells were cultured in Dulbecco’s Modified Eagle Medium (DMEM) supplemented with 10% fetal bovine serum in the incubator of 5% CO_2_ and 37℃.

### Preparation of hAMSCs conditioned medium (hAMSCs-CM)

hAMSCs were seeded in 10-cm plates at 60% density. A serum-free medium was used to continuously culture the cells for 48 h after confluence reached 80%. The concentration was collected and centrifuged in an ultrafiltration centrifuge tube at 3500 g/h for 1 h and then stored in the refrigerator at -80℃.

### Quantitative real-time polymerase chain reaction(qPCR)

Total RNA was isolated with an RNAiso Plus reagent kit (Takara, Japan) according to the manufacturer’s instructions. cDNA was generated by reverse transcription of.

RNA using Prime-Script RT reagent kit (Takara, Japan). The qPCR reaction system was listed with specific primers in Table 1 and SYBR Premix Ex Taq II (Takara, Japan). The mRNA level was calculated by the 2^−ΔΔCt^ method, and GAPDH was used as the endogenous control.

**Table 1:** The primers sequence used for qPCR

Gene	Forward primer (5′-3′)	Reverse primer (5′-3′)
GAPDH	CATCATCCCTGCCTCTACTGG	GTGGGTGTCGCTGTTGAAGTC
Cytokeratin19	AACGGCGAGCTAGAGGTGA	GGATGGTCGTGTAGTAGTGGC
E-cadherin	CGAGAGCTACACGTTCACGG	GGGTGTCGAGGGAAAAATAGG
Vimentin	GACGCCATCAACACCGAGTT	CTTTGTCGTTGGTTAGCTGGT
Collagen I	GAGGGCCAAGACGAAGACATC	CAGATCACGTCATCGCACAAC
Fibronectin	CGGTGGCTGTCAGTCAAAG	AAACCTCGGCTTCCTCCATAA

### Western blotting (WB)

Total protein was extracted from tissues and cells with RIPA lysate (P0013B, Beyotime) mixed with PMSF (ST506, Beyotime) and was quantified using the Enhanced BCA Protein Assay Kit (P0010, Beyotime). The protein was loaded in the lanes, separated by 7.5% SDS-PAGE, and transferred to the polyvinylidene difluoride membrane. After blocking with 5% non-fat milk, the membrane was incubated at 4℃ overnight with primary antibodies against Vimentin (GB11192, dilution 1:1000; Servicebio), Cytokeratin19 (CK19, 10712-1-AP, dilution 1:5000; Proteintech), E-cadherin (E-cad, 20874-1-AP, dilution 1:5000; Proteintech), Collagen I (67288-1-Ig, dilution 1:5000; Proteintech), Fibronectin (FN, 15613-1-AP, dilution 1:5000, Proteintech), GAPDH (60004-1-Ig, dilution 1:5000; Proteintech). The membrane was incubated with HRP-conjugated secondary antibodies (SA00001-1, dilution 1:5000, Proteintech or SA00001-2, dilution 1:5000; Proteintech) for; 1 h at room temperature. The Omni-ECL TMFemto Light Chemiluminescence Kit (Epizyme) was used to capture blot signals. Image J software was used to quantify at least three independent experiments for the western blot bands, and GAPDH was used as the endogenous control.

### Immunofluorescence staining (IF)

hAMSCs were seeded in six-well plates with mounted slides and co-cultured with IUA-THESCs. hAMSCs were harvested and fixed with 4% paraformaldehyde for 20 min, treated with 0.5%TritonX-100 for 10 min, and sealed with 5% BSA for 30 min. The samples were incubated overnight at 4℃ with the primary antibodies Vimentin (GB11192, dilution 1:500; Servicebio), CK19 (10712-1-AP, dilution 1:500; Proteintech), and E-cad (20874-1-AP, dilution 1:200; Proteintech). Cells were then incubated with secondary antibodies (A0516, dilution 1:500, Cy3-labeled Goat Anti-Rabbit IgG (H + L); Beyotime) for 1 h at RT. Nuclei were counterstained with DAPI (AR1176;Boster) for 5 min at RT. The cells were observed by fluorescence orthotopic microscope after being dripped with antifade mounting medium and sealed with slides.

### Establishment of IUA rat model

All animal experiment protocols were approved by the Ethics Committee of Chongqing Medical University (No. IACUC-CQMU-2023-0373). Sprague–Dawley (SD) female rats weighing 200–220g were purchased from the Animal Experimental Center of Chongqing Medical University and raised in a controlled environment at 22ºC with a 12 h/12 h light/dark cycle. Rats in diestrus were selected based on the vaginal smear analysis and anaesthetized with 5% chloral hydrate anaesthesia (MACKLIN) (10 ml/kg) by intraperitoneal injection. The abdomen was opened along the median of the lower abdomen, and the bilateral uterine horns were exposed. The uterine ends were ligated with 5 − 0 silk sutures and 95% ethanol was injected to fill the uterine cavity and maintained for 2 min. The abdominal incision was sutured after being washed with saline two times. Only incision and closure steps were performed in the sham group.

### Transplantation of hAMSCs and hAMSCs-CM

Each group performed different treatment measures two weeks after establishing the IUA rat model. Only saline was injected into the uterine cavity in the sham group. No treatment was given in the IUA group. A 100ul cell suspension containing 1 × 10^7^ hAMSCs and 100ul conditioned medium was injected into the uterine cavity in hAMSCs and hAMSCs-CM groups. Besides, 1 × 10^7^ of hAMSCs, combined with 100ul polyethylene glycol citrate-co-N-isopropylacrylamide gelatin (PPCNg), were injected into the uterine cavity and vagina, respectively, in the PPCNg/hAMSCs group.

### H&E and MASSON staining

The uterine specimens of rats were collected at different times, fixed with 4% paraformaldehyde and sliced. H&E and Masson staining were performed according to the manufacturer’s instructions (Solarbio). The number of glands and thickness of the endometrium were revealed by H&E staining. The fibrotic area was shown by Masson staining. Image J software was applied to analyze the images.

### Immunohistochemical staining (IHC)

The prepared paraffin sections were deparaffinized, hydrated, antigen recovered and incubated with 3% hydrogen peroxide solution and 10% goat serum at 37 ℃ for 30 min to block endogenous peroxidase activity and remove non-specific binding. It was incubated overnight for 16 h at 4 °C with the primary antibody for CK19 (10712-1-AP, dilution 1:5000; Proteintech), E-cad (20874-1-AP, dilution 1:5000; Proteintech), Collagen I (67288-1-Ig, dilution 1:5000; Proteintech), FN (15613-1-AP, dilution 1:5000; Proteintech) and subsequently incubated with biotin-labeled secondary antibody for 30 min. The colour reaction was developed with diaminobenzidine (DAB) (ORIgene), and nuclei were stained with hematoxylin (Solarbio). Image J was used to measure the percentage of positive staining area.

### Statistical analysis

Statistical analysis was performed with GraphPad Prism 9. The data were presented as mean ± standard deviation (SD) from at least three independent experiments. Group comparisons were determined by one-way analysis of variance (ANOVA) followed by Tukey’s post hoc test. GAPDH was used as the endogenous control. *P* < 0.05 was considered statistically significant. (Ns indicates no significance, *indicates *P* < 0.05, **indicates *P* < 0.01, ***indicates *P* < 0.001, ****indicates *P* < 0.0001).

## Results

### hAMSCs reduced the expression of fibrosis-related markers in IUA-THESCs after co-culture for two days via paracrine

**Fig. 1:**
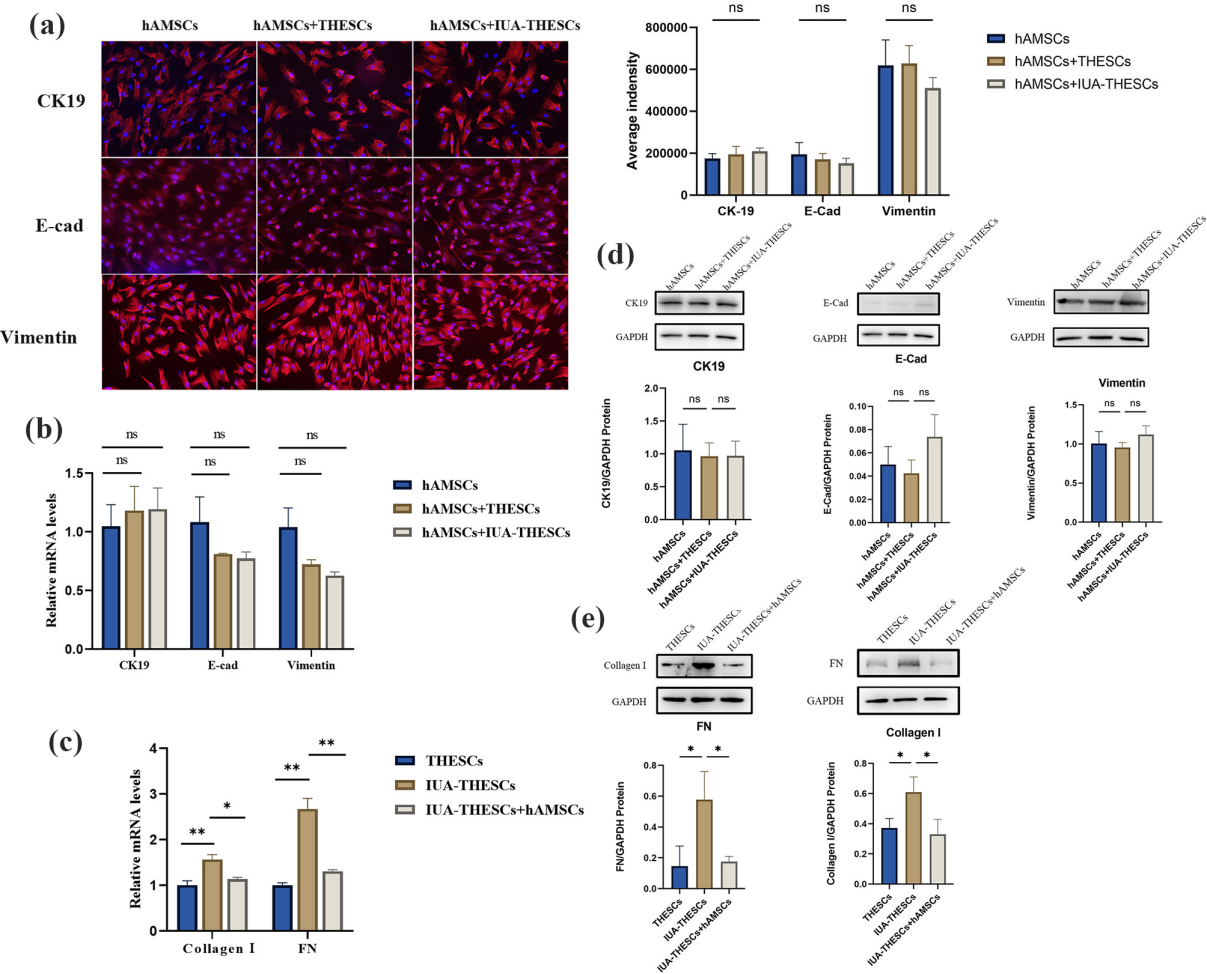
hAMSCs treated IUA by paracrine in vitro for 2 days. **(a)** The expression of CK19, E-cad and Vimentin were detected by IF. (scale bar = 50 μm). **(c, e)** qPCR and WB were used to detect the expression of Collagen I and FN in THESCs treated by hAMSCs. **(b, d)** The levels of CK19, E-cad and Vimentin in IUA-THESCs treated by hAMSCs were detected by qPCR and WB. Quantification results normalized to GAPDH are shown as mean ± SD (*n* = 3), one-way ANOVA test. Ns indicates no significance, *P < 0.05, **P < 0.01

The IUA-THESCs cell model was constructed by intervening THESCs with TGF-β1 in vitro. To verify that hAMSCs can reduce the expression of fibrosis-related markers of FN and collagen I in IUA-THESCs, we conducted a transwell co-culture study of hAMSCs and IUA-THESCs, which was designed to block the direct contact between the two types of cells. qPCR and WB detected collagen I and FN expression levels in IUA-THESCs. The results revealed that mRNA levels of the two marker genes in IUA-THESCs were significantly down-regulated after two days of co-culture with hAMSCs (Fig. 1. c), and the protein levels detected by WB were consistent with qPCR (Fig. 1. e). These results indicated that hAMSCs could effectively reduce fibrosis-related expression in IUA-THESCs after co-culture for 2 days via paracrine.

Further, to clarify the expression changes of hAMSCs after 2 days of co-culture with IUA-THESCs, to demonstrate whether hAMSCs showed a trend of epithelial transformation. IF was used to detect the epithelial marker genes levels of CK19 and E-cad and the levels of Vimentin stroma marker genes. The results showed that compared with isolated hAMSCs, there was no significant difference in the expression of these marker genes in hAMSCs co-cultured for two days (Fig. 1. a). Subsequently, mRNA and protein levels of the above marker genes were quantitatively detected by qPCR and WB, and the results were consistent with cellular IF (Fig. 1. b, d).

This finding highlights that hAMSCs inhibited fibrosis through the paracrine pathway at 2 days of co-culture. However, they did not show a tendency to differentiate into epithelial cells at this time.

### hAMSCs exhibited differential expression of epithelial and stroma-related markers after co-culture for 6 days

**Fig. 2:**
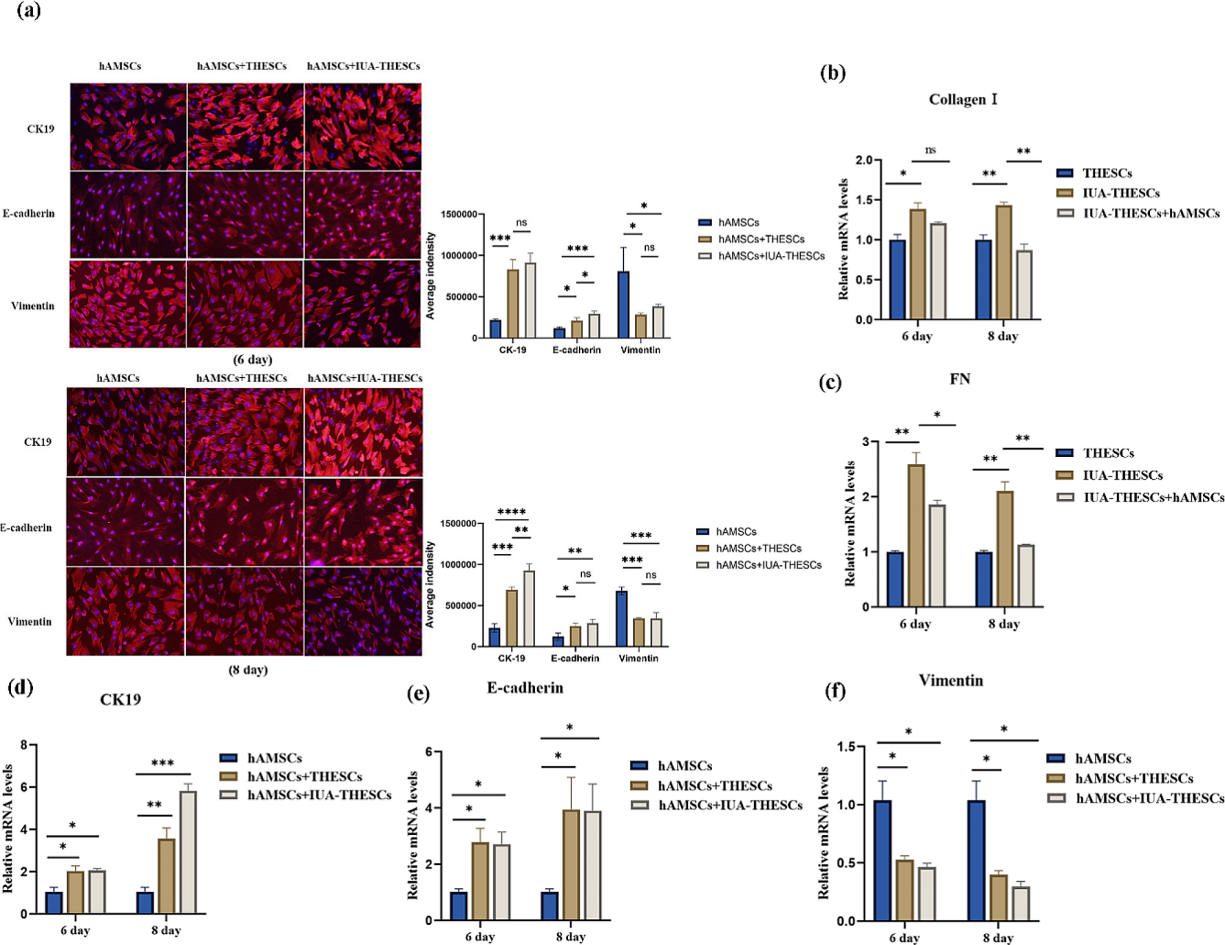
hAMSCs showed a tendency for epithelial transformation. **(a)** The expressions of CK19, E-cad and Vimentin in IUA-THESCs on the 6 and 8 days of hAMSCs treatment were detected by IF. (scale bar = 50 μm). **(d, e, f)** The expression of CK19, E-cad and Vimentin in IUA-THESCs on days 6 and 8 of hAMSCs treatment were detected by qPCR and WB. **(b, c)** The expressions of collagen I and FN in IUA-THESCs on the 6 and 8 days of hAMSCs treatment were quantitatively detected. Quantification results normalized to GAPDH are shown as mean ± SD (*n* = 3), one-way ANOVA test. Ns indicates no significance, *P < 0.05, **P < 0.01, ***P < 0.001, ****P < 0.001

Previous studies have reported that hAMSCs could differentiate into endometrial epithelial cells [12]. However, the above results do not support this conclusion, which may be related to insufficient co-culture time. Therefore, we extended the co-culture time to reproduce both paracrine and transdifferentiation of hAMSCs in the same co-culture system. The results showed that after 6 days of co-culture with IUA-THESCs, the expressions of CK19 and E-cad in hAMSCs significantly increased while the levels of Vimentin decreased. Subsequently, we extended the co-culture time to 8 days, with no statistical difference from the results of 6 days. Noticeably, after co-culture for 8 days, we found that the morphology of hAMSCs did not change significantly, indicating that hAMSCs only showed a tendency to transform into epithelial cells at the molecular level rather than already differentiated into renal epithelial cells, which may require a longer co-culture times (Fig. 2).

In order to determine whether the co-culture system of IUA-THESCs and hAMSCs can promote the transdifferentiation process of hAMSCs compared with the co-culture system of normal THESCs and hAMSCs, our study compared the expression levels of CK19, E-cad and Vimentin of hAMSCs in the two groups at 2, 6 and 8 days of co-culture, respectively. The difference was not statistically significant. These findings suggested that hAMSCs tended to transit into epithelial cells after 6 days of co-culture with THESCs. IUA-THESCs were not necessary to induce this tendency in hAMSCs (Fig. 2).

### hAMSCs-CM promoted endometrium remodeling in vivo

**Fig. 3:**
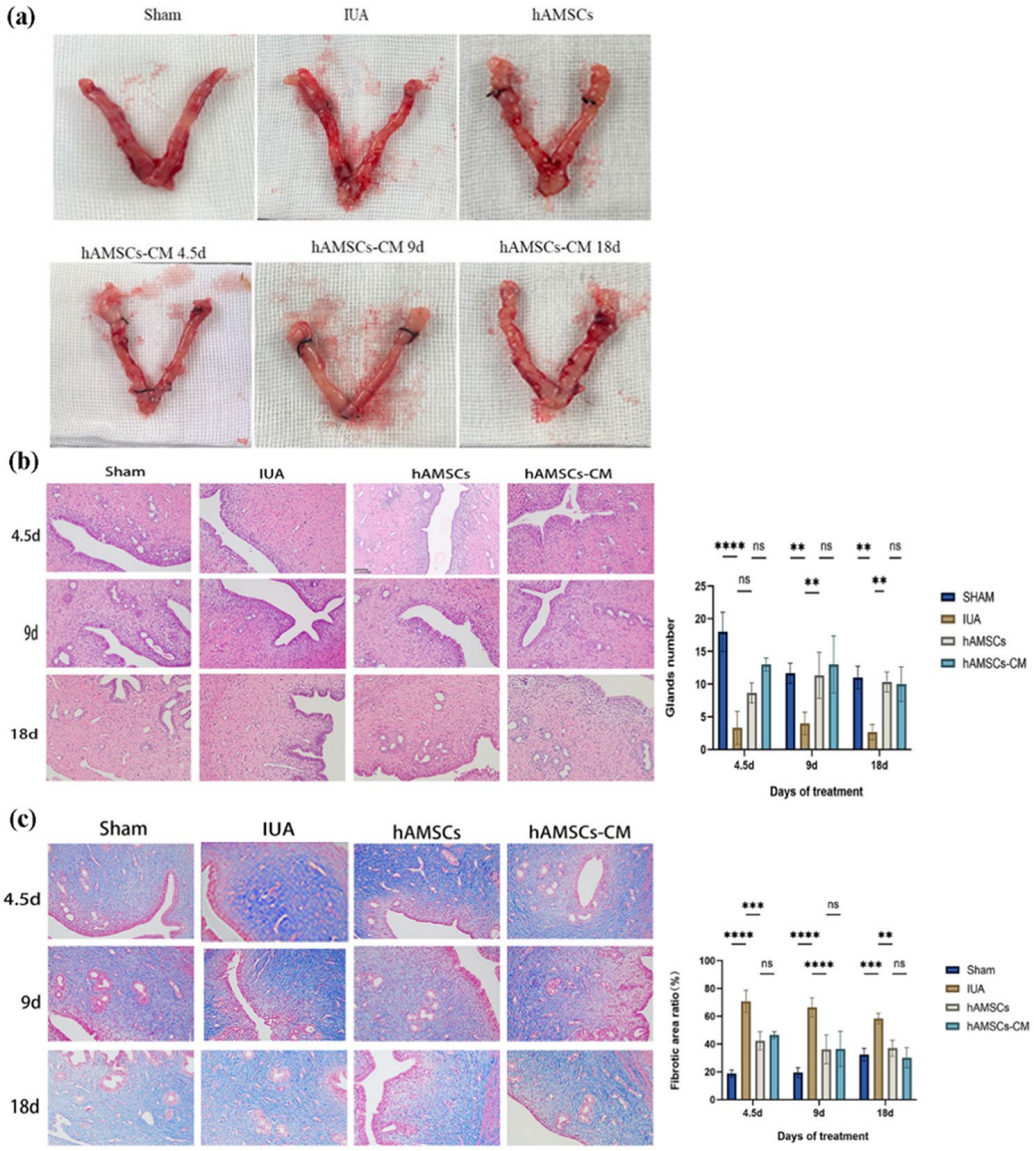
hAMSCs-CM inhibited endometrial fibrosis and promoted structural remodeling **(a)** The shape of uterus after hAMSCs-CM treatment. **(b)** H&E staining was used to evaluate endometrial regeneration and repair in IUA rats (scale bar = 50 μm). **(c)** MASSON staining was used to assess the area reduction of endometrial fibrosis in IUA rats (scale bar = 50 μm). The results are shown as mean ± SD (*n* = 3), one-way ANOVA test. Ns indicates no significance, **P < 0.01, ***P < 0.001, ****P < 0.0001

To confirm that hAMSCs reduced endometrial fibrosis and promoted endometrium remodeling through paracrine in vivo, hAMSCs-CM contained all components secreted by hAMSCs were collected and injected into the uterine cavity of IUA rats. Endometrial thickness and number of glands were assessed by H&E staining, and fibrosis area was assessed by MASSON staining. The results showed that in the first estrus cycle (4.5d) after hAMSCs-CM treatment, the number of glands increased significantly while the fibrosis area of endometrial decreased significantly. Remarkably, there was no statistical significance between the hAMSCs-CM and hAMSCs groups. In addition, after 9 and 18 days of treatment with hAMSCs-CM, the effects of remodeling uterine structure and epithelial repair remained stable, and there was still no statistical difference compared with the hAMSCs group (Fig. 3. b, c). In addition, the shape of uterus after hAMSCs-CM treatment was significantly recovered compared with that in IUA group which was stiff and thin (Fig. 3.a).

### hAMSCs-CM facilitated the regeneration of endometrium

**Fig. 4:**
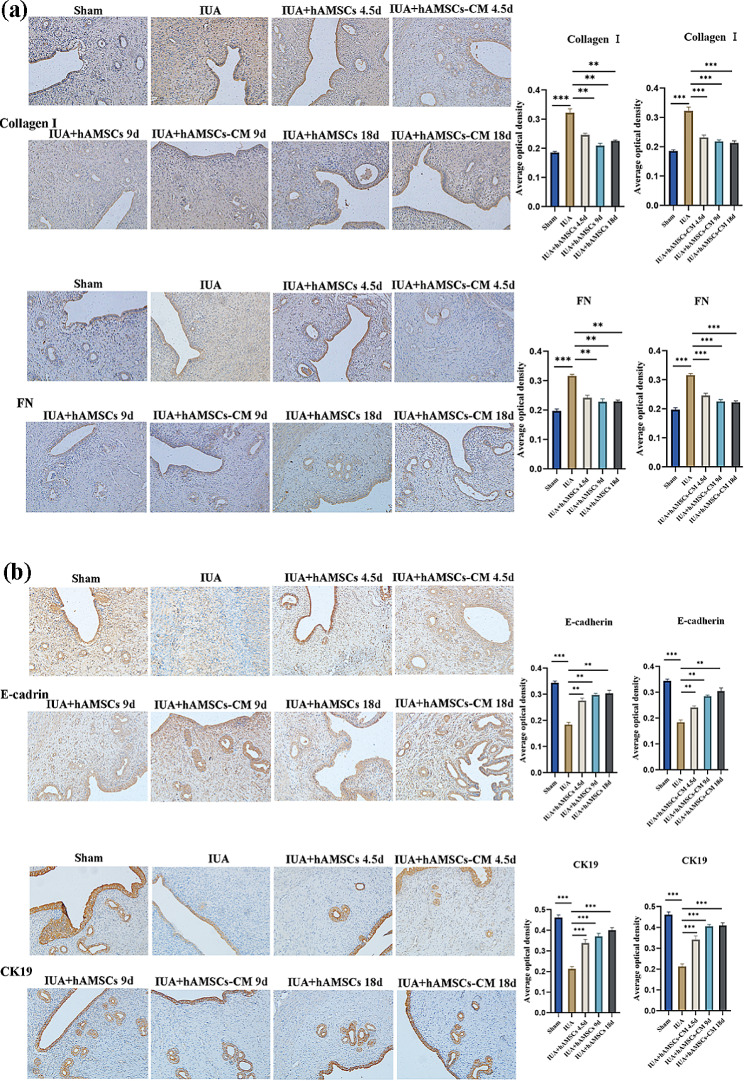
hAMSCs-CM suppressed fibrosis and promotes epithelial regeneration. **(a)** IHC was used to detect the level of fibrosis marker protein after hAMSCs-CM treatment in IUA rats (scale bar = 50 μm). **(b) IHC** was used to detect endometrial epithelial cytoskeleton protein levels after hAMSCs-CM treatment in IUA rats (scale bar = 50 μm). The results are shown as mean ± SD (*n* = 3), one-way ANOVA test. **P < 0.01, ***P < 0.001

The expressions of collagen I and FN are closely related to the pathological process of IUA, and CK19 and E-cad are mainly cytoskeletal proteins that maintain the integrity of endometrial epithelial cells. IHC was used to detect the protein levels of epithelial and fibrotic markers. The results suggested that epithelial-related protein of CK19 and E-cad increased significantly (Fig. 4. a), and fibrosis marker protein of collagen I and FN decreased significantly after hAMSCs-CM treatment (Fig. 4. b), consistent with MASSON and H&E staining results. These results manifested that hAMSCs-CM has the same efficacy as hAMSCs in inhibiting fibrosis and promoting endometrial repair, supporting that hAMSCs promoted endometrial remodeling through paracrine in vivo.

### The effect of hAMSCs by vaginal injection was comparable to that by intrauterine injection

**Fig. 5:**
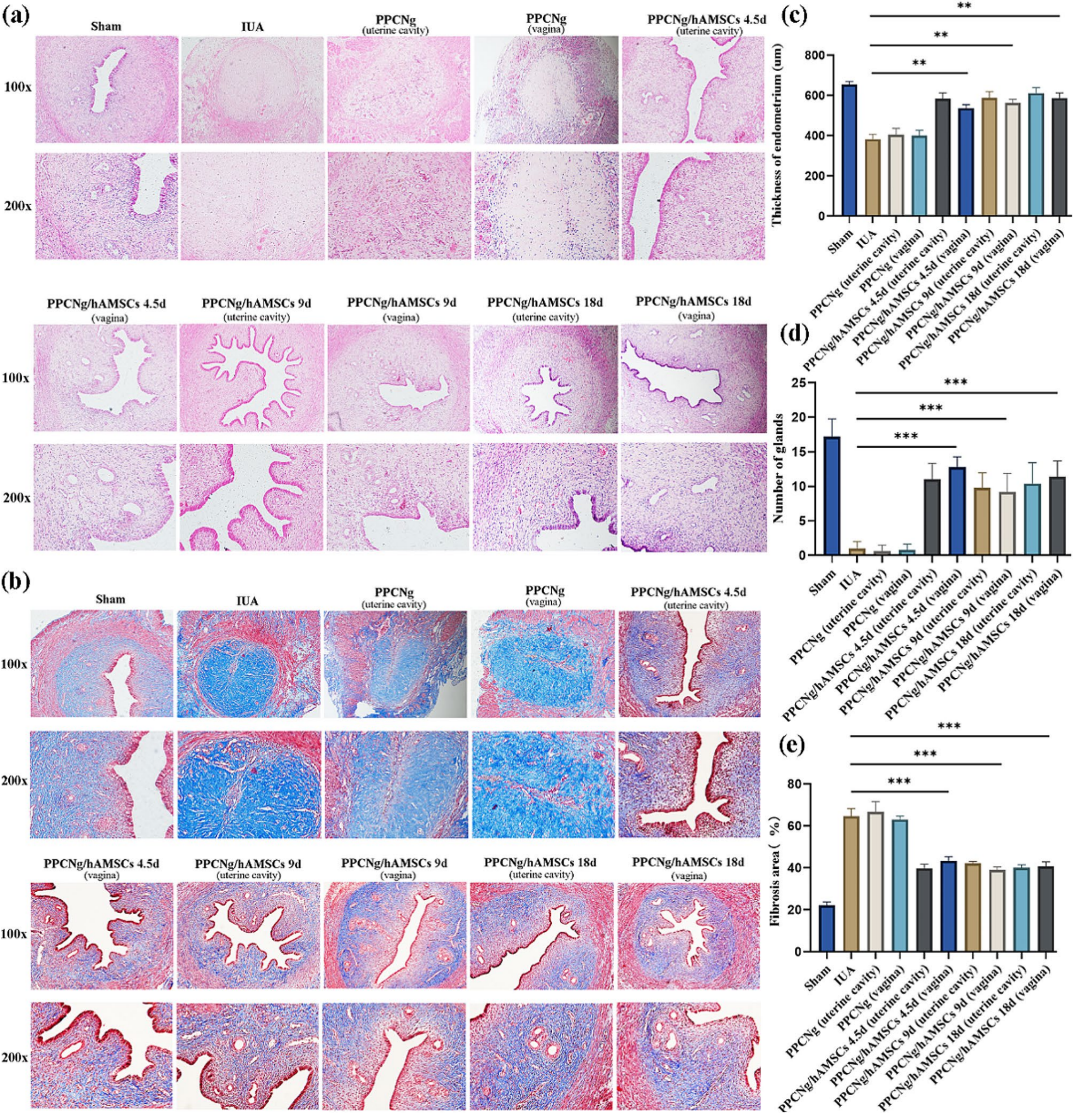
Vaginal injection of hAMSCs was also effective in treating IUA. **(a, c, d)** H&E staining was used to compare the effect of hAMSCs vaginal injection and intrauterine injection on endometrial repair (bar = 50 μm and 100 μm). **(b, e)** MASSON staining was used to compare the effect of hAMSCs vaginal injection and intrauterine injection on the reduction of endometrial fibrosis (bar = 50 μm and 100 μm). The results are shown as mean ± SD (*n* = 3), one-way ANOVA test. **P < 0.01, ***P < 0.001

The vagina is the preferred method of medicine or delivery for females, which is non-invasive, safe and convenient. In our previous studies, PPCNg is a thermo-responsive biomaterial, and it was proved to increase hAMSCs adhesion, supporting cell survival and implantation [17]. In this study, hAMSCs combined with PPCNg were administered by vaginal injection and intrauterine injection, respectively, to verify the efficacy of the vaginal delivery route and reconfirm the paracrine role of hAMSCs in vivo. H&E and MASSON staining were used to evaluate endometrial glandular hyperplasia and fibrotic area. The results showed that after 4.5 days of hAMSCs treatment, endometrial thickness and the number of glands in the vaginal injection group significantly increased, and the fibrosis area was significantly reduced, with no statistical difference compared with the intrauterine group (Fig. 5).

**Fig. 6:**
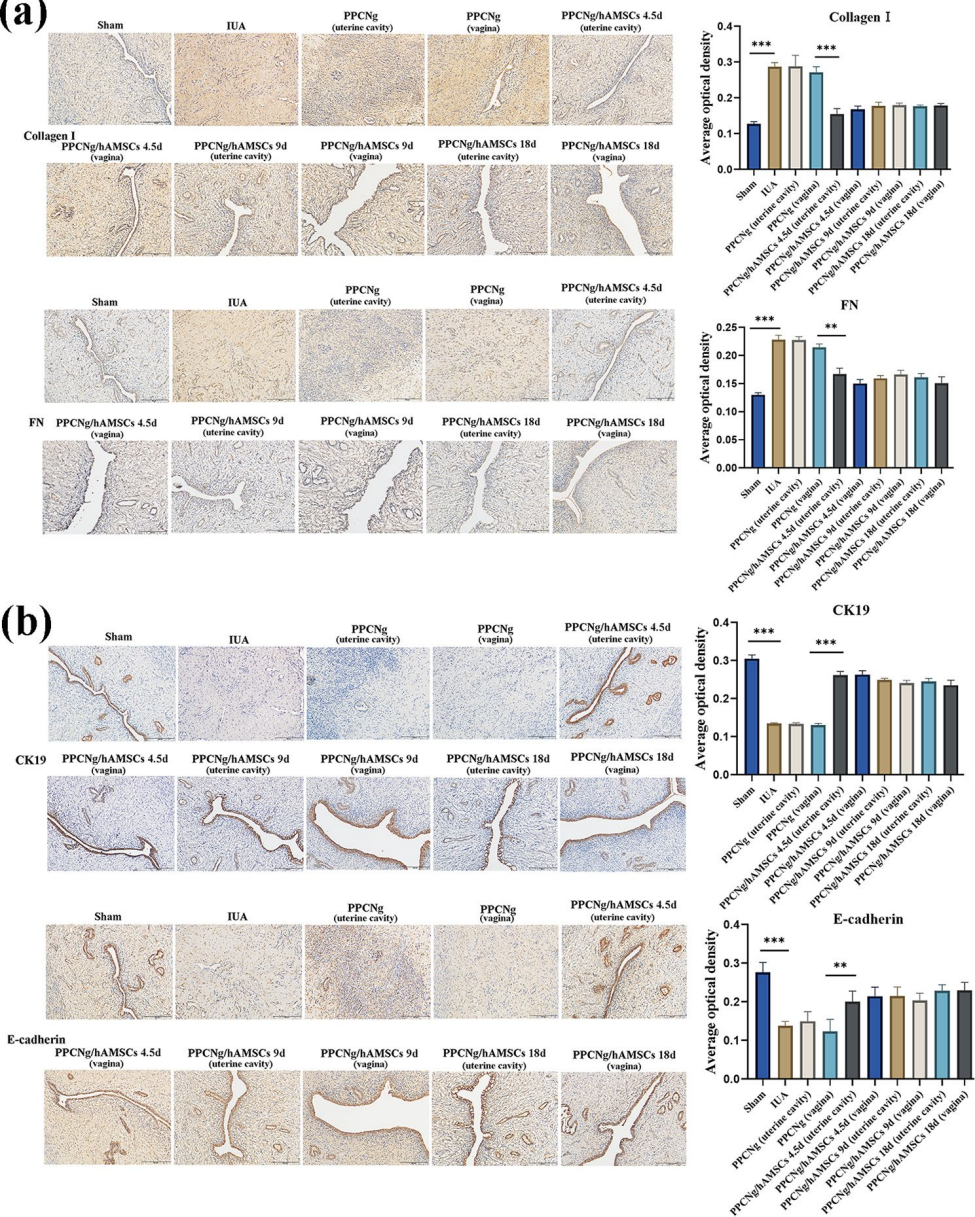
Vaginal injection of hAMSCs, effectively promotes endometrial regeneration. **(a)** The levels of collagen I and FN were detected by IHC after vaginal injection and intrauterine injection of hAMSCs (bar = 50 μm). **(b)** IHC was used to compare the expression of epithelial-associated proteins after vaginal injection and intrauterine injection of hAMSCs. (bar = 50 μm). The results are shown as mean ± SD (*n* = 3), one-way ANOVA test. **P < 0.01, ***P < 0.001

In addition, IHC was used to detect the expression levels of epithelial marker proteins and fiber-associated proteins. The results showed that CK19 and E-cad protein levels of endometrium in the vagina group significantly increased (Fig. 6. a). At the same time, the expressions of collagen I and FN significantly decreased, with no statistically significant differences compared with the intrauterine group (Fig. 6. b). These findings above indicated that vaginal injection of hAMSCs could achieve the same therapeutic effect as intrauterine injection, which was an effective and convenient drug delivery method. They reconfirmed that hAMSCs exerted biological effects through paracrine cytokines absorbed into the blood via the vaginal wall.

### GFP-hAMSCs continuously colonized the endometrial stroma in vivo

**Fig. 7:**
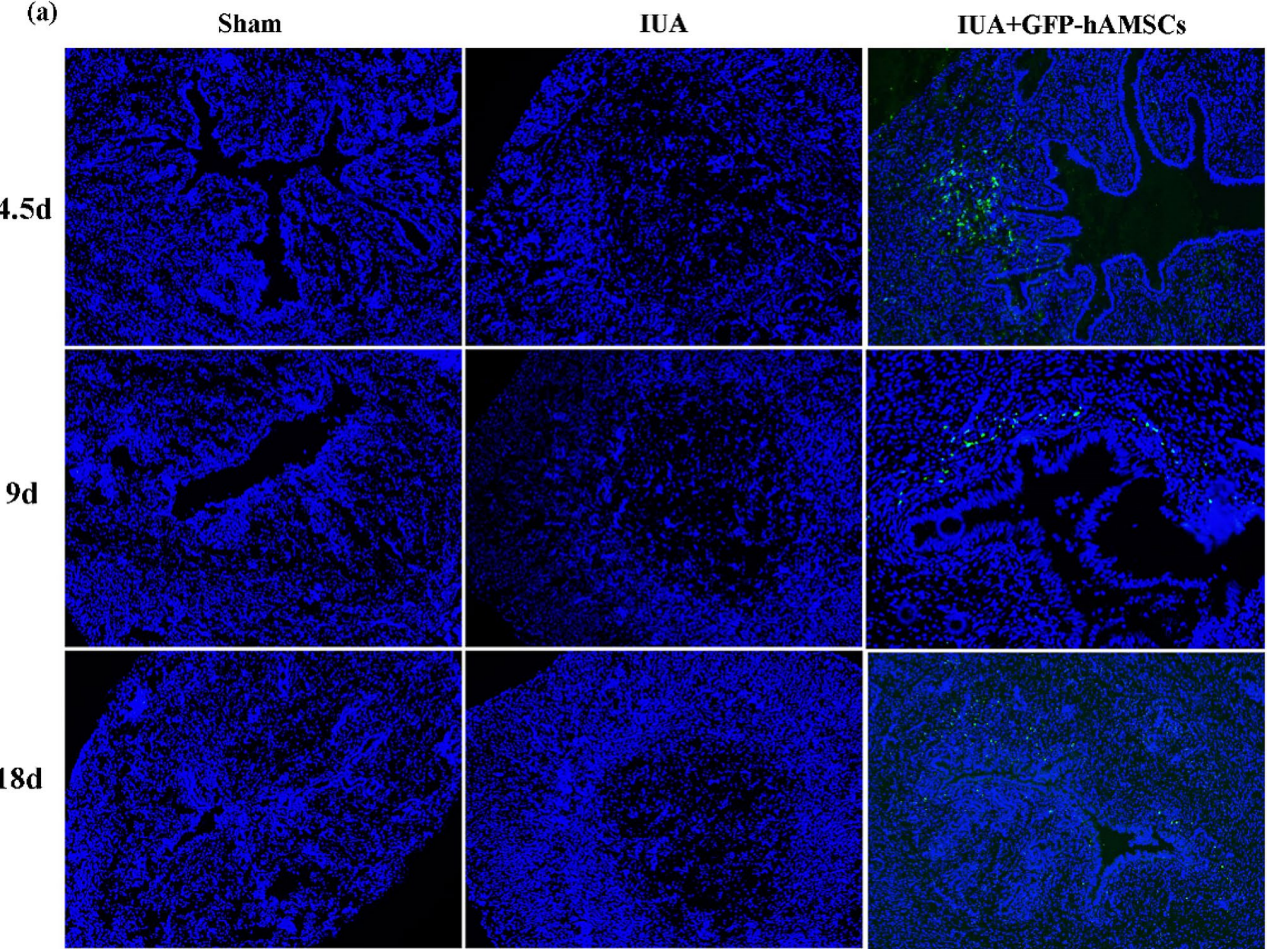
GFP-hAMSCs colonized the endometrial stroma in vivo. **(a)** IF showed the expression and localization of GFP-labeled hAMSCs in IUA rats (bar = 50 μm)

To determine whether hAMSCs transdifferentiated into epithelial cells in vivo, GFP was used to label hAMSCs stably for tracking and localization. GFP-hAMSCs were injected into the uterine cavity of IUA rats. The results showed that the damaged endometrium was effectively repaired at 4.5 days of hAMSCs treatment, while GFP-hAMSCs colonized the endometrial stroma instead of the epithelium, indicating that hAMSCs inhibited fibrosis and repaired the endometrium even though they were not transformed into epithelial cells. Subsequently, we extended the treatment time to see if hAMSCs transformed into epithelial cells in vivo. The results showed that the fluorescence intensity of GFP decreased significantly at 9 days and 18 days, but it was always colonized in the endometrial stroma rather than the epithelium (Fig. 7).

These results indicated that hAMSCs gradually underwent apoptosis in vivo and did not successfully transform into epithelial cells before apoptosis. This supports the idea that its therapeutic effect on damaged endometrium was mainly related to paracrine cytokines.

## Discussion

IUA is a critical factor of infertility in women of childbearing age. The primary.

objective of IUA therapy is to optimize fertility outcomes. However, the efficacy of the current treatment is minimal, and it is urgent to determine a safe and effective approach to manage IUA clinically.

A randomized controlled clinical study was previously conducted by our team on fresh amniotic membrane transplantation in 100 patients with IUA after TCRA. The recurrence rate was significantly reduced [18]. However, unfortunately, there are risks such as shedding and infection in application. Therefore, hAMSCs extracted from the fresh amniotic membranes were then used to treat IUA and were found to effectively promote endometrial regeneration and improve fertility [17], but the exact mechanism remained unclear.

The secretory factors produced by MSCs, such as growth factors, extracellular vesicles and exosomes, were critical in improving the local microenvironment and promoting tissue repair and maintenance [8, 19–22]. Qi et al. reported that the administration of menstrual blood-derived stromal cells-derived exosomes achieved similar therapeutic effects to MSC transplantation, including alleviating endometrial fibrosis and improving fertility in IUA rats [23]. Liu et al. illustrated that exosomes derived from placental mesenchymal stem cells can repair endometrial damage and enhance fertility by regulating the TGF-β/Smad pathway [24]. Li et al. also confirmed in vitro that hAECs effectively treated IUA-THESCs through paracrine effects by coculturing hAECs and IUA-THESCs [15]. In this study, a coculture system of hAMSCs and IUA-THESCs was constructed to simultaneously verify the effects of paracrine and transdifferentiation. The results showed the expression reduction of fibrosis-related proteins in hAMSCs after co-culture for 2 days. Furthermore, the injection of hAMSCs-CM into the uterine cavity of IUA rats significantly reduced the fibrosis area of the endometrium and promoted the proliferation of endometrial and epithelial glands. These results manifested that hAMSCs treated IUA effectively via paracrine.

Single-cell RNA sequencing (scRNA-seq) was applied to explore the cell differentiation trajectory after hUCB-MSCs transplanted into IUA endometrium by Hua et al. [13], indicating that the transplanted hUCB-MSCs transdifferentiated into endometrial cells: epithelial, fibroblast and macrophage. Despite strong evidence that cells translated into epithelial cells, it was still doubtful whether the transplanted cells were sufficient to replace the loss of intimal epithelial cells and whether the stem cells with differentiation capacity mainly exerted biological effects through transdifferentiation. Therefore, this study extended the co-culture time to induce transdifferentiation in vitro. The result showed that it took 6 days for the changes in markers of epithelial and stroma of hAMSCs, and no difference in morphology of hAMSCs was observed under the microscope even after 8 days of co-culture. It indicated that hAMSCs have not yet wholly transdifferentiated into epithelial cells of the endometrium but only showed a tendency of transformation at the molecular level. These results highlight that the transdifferentiation of hAMSCs took more than 6 days, far exceeding the 2 days required for paracrine effects.

To prove that damaged endometrium was repaired by paracrine of hAMSCs in vivo, the IUA rats were successfully established, and the repair efficacy of damaged endometrium in the hAMSCs-CM group and isolated hAMSCs group was compared. In addition, the colonization site of hAMSCs in vivo was tracked by the fluorescence produced by GFP to determine whether it was transformed into epithelial cells. The results showed that hAMSCs-CM exerted the same effect as hAMSCs on the injured endometrium, and hAMSCs continuously colonized the endometrial stroma rather than the epithelium. With the extension of transplantation time, the fluorescence intensity of GFP-hAMSCs gradually decreased, suggesting the existence of apoptosis. Xin et al. found that the transplanted MSCs in vivo gradually disappeared due to apoptosis and clearance of host cells around the seventh day [25], which was consistent with our outcomes. Overall, no direct evidence was found in our study that hAMSCs transdifferentiated into epithelial cells in vivo, supporting that hAMSCs created an appropriate environment for restoration via paracrine in the early stages of endometrial injury.

MSCs were transplanted in different routes, including tail vein injection, intraperitoneal injection, and especially intrauterine injection, to promote endometrium repair. In contrast, the vaginal administration is more convenient and non-invasive. Moreover, due to the obstruction of MSCs entering the blood through the vaginal squamous epithelium, the therapeutic effect of MSCs in the vagina was achieved by the absorption of active ingredients produced by paracrine. Kong et al. [26] found that the vaginal application of L. crispatus-pMG36e-mCXCL12 strains significantly diminished the levels of IL-β1 and TNF-αin serum and uterine tissues of IUA mice and resulted in the inhibition of the inflammatory and fibrotic signal pathways in the uterine tissues. These results suggested that vaginal administration was safe and feasible. In this study, MSCs were combined with the thermosensitive material PPCNg to enhance vaginal colonization. The results showed that vaginal injection of hAMSCS resulted in increased endometrial glands and decreased fibrosis area in IUA rats, which was almost equivalent to intrauterine injection. In addition to the effectiveness of vaginal administration, hAMSCs were repeatedly demonstrated to act on damaged endometrium via paracrine potent factors.

In summary, our research confirmed that hAMSCs ameliorate endometrial fibrosis of IUA via paracrine, preferentially than transdifferentiation, providing the latest insights into the precision treatment of IUA with hAMSCs and a theoretical basis for promoting the “cell-free therapy” of MSCs.

### Electronic supplementary material

Below is the link to the electronic supplementary material.

**Supplementary Material 1:** Figure S1

**Supplementary Material 2:** CK19

**Supplementary Material 3:** Collagen I

**Supplementary Material 4:** E-cad

**Supplementary Material 5:** FN

**Supplementary Material 6:** Vimentin

## Data Availability

No datasets were generated or analysed during the current study.

## Abbreviations

CK19: Cytokeratin19
FN: Fibronectin
hAECs: Human amniotic epithelial cells
hAMSCs: Human amniotic mesenchymal stem cells
hAMSCs-CM: Human amniotic mesenchymal stem cells conditional medium
hUCB-MSCs: Human umbilical cord blood-derived MSCs
IUA: Intrauterine adhesion
IUD: Intrauterine device
MSCs: Mesenchymal stem cells
PPCNg: Polyethene glycol citrate-co-N-isopropylacrylamide gelatin
SD: The Sprague-Dawley rats
TCRA: Hysteroscopic transcervical resection of adhesion
TGF-β: Transforming growth factor beta
THESCs: Transformed human endometrial stromal cells
