# Correction: Human amnion mesenchymal stem cells promote endometrial repair via paracrine, preferentially than transdifferentiation

**DOI:** 10.1186/s12964-024-01706-7

**Published:** 2024-06-13

**Authors:** Xiyue Huang, Xiao Yang, Jinglin Huang, Ling Wei, Yanhua Mao, Changjiang Li, Yingfeng Zhang, Qiuhong Chen, Shasha Wu, Lele Xie, Congcong Sun, Wenwen Zhang, Jia Wang

**Affiliations:** Department of Obstetrics and Gynecology, The University-Town Hospital of Chongqing Medical University, No. 55, Daxuecheng Middle Road, Chongqing, 401331 China

**Correction: Cell Commun Signal 22, 301 (2024)**

10.1186/s12964-024-01656-0

Following the publication of original article [1], the authors noticed a typesetting error, whereby the note to Fig. 3 was mistakenly omitted. The note to the figure is as follows:

(**a**) The shape of uterus after hAMSCs-CM treatment. (**b**) H&E staining was used to evaluate endometrial regeneration and repair in IUA rats (scale bar = 50 μm). (**c**) MASSON staining was used to assess the area reduction of endometrial fibrosis in IUA rats (scale bar = 50 μm). The results are shown as mean ± SD (*n* = 3), one-way ANOVA test. Ns indicates no significance, **P < 0.01, ***P < 0.001, ****P < 0.0001.

The original article [1] has been updated.
