# Supplementary material for: Human amnion mesenchymal stem cells promote endometrial repair via paracrine, preferentially than transdifferentiation
Source: Cell Commun Signal. 2024 May 31;22:301. doi: 10.1186/s12964-024-01656-0 (PMC11140932; doi:10.1186/s12964-024-01656-0)
Supplement: Supplementary file 1 — Supplementary Material 1: Figure S1 [file 12964_2024_1656_MOESM1_ESM.pdf]

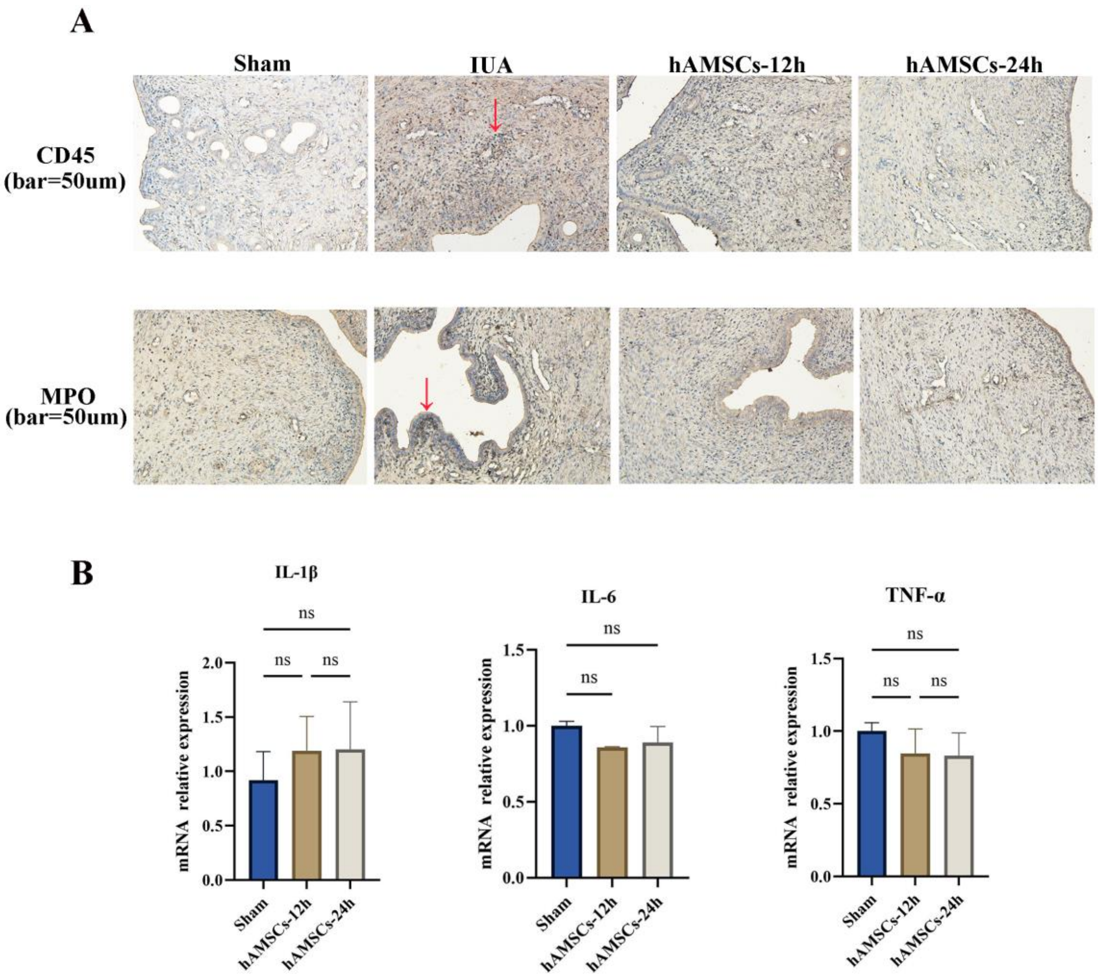

Fig.S1 The expression of inflammation-related factors after injection of hAMSCs into uterus of SD rats. **(A)** IHC showed the expression of MPO and CD45 in the uterus (scale bar = 50um). **(B)** qPCR detected the expression of IL-1, IL-6, and TNF-α. Quantification results normalized to GAPDH are shown as mean ± SD (n = 3), a one-way ANOVA test. Ns indicates no significance.
