# Supplementary figures and images for: Human amnion mesenchymal stem cells promote endometrial repair via paracrine, preferentially than transdifferentiation
Source: Cell Commun Signal. 2024 May 31;22:301. doi: 10.1186/s12964-024-01656-0 (PMC11140932; doi:10.1186/s12964-024-01656-0)

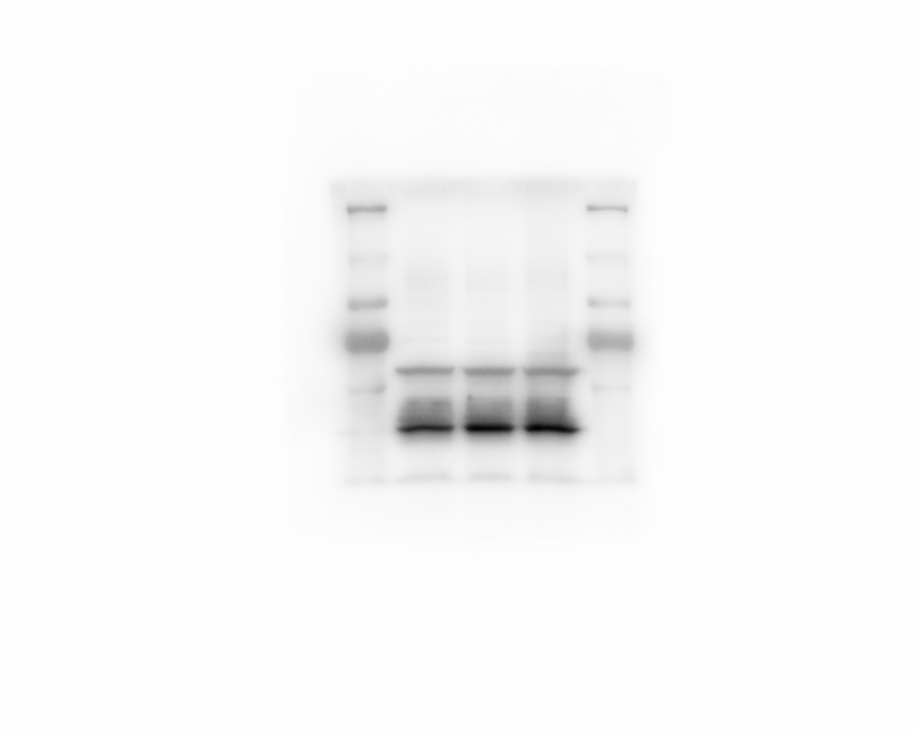

Supplement: Supplementary file 2 — Supplementary Material 2: CK19 [file 12964_2024_1656_MOESM2_ESM.zip › CK19/CK19 -2 _20240130_192628_00.00_8bit(2).tif]

CK19-1

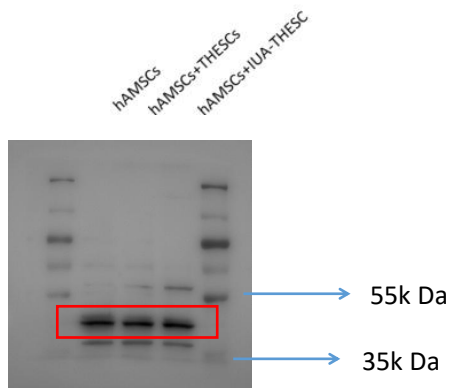

GAPDH-1

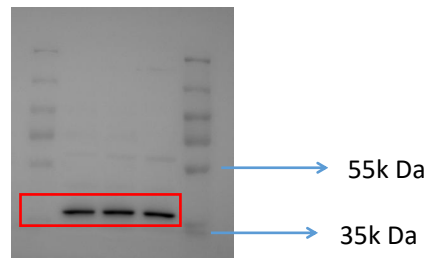

CK19-2

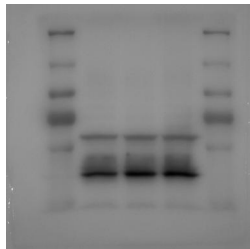

GAPDH-2

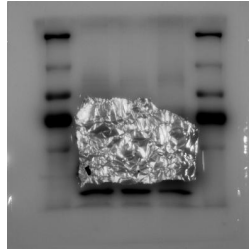

CK19-3

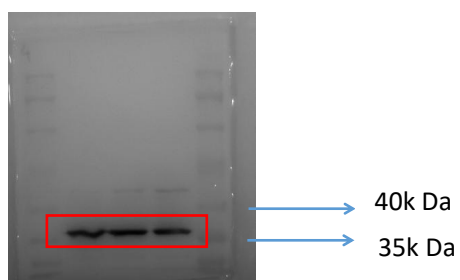

GAPDH-3

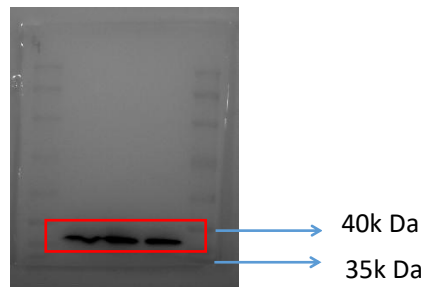

(different marker)

Supplement: Supplementary file 2 — Supplementary Material 2: CK19 [file 12964_2024_1656_MOESM2_ESM.zip › CK19/CK19.pdf]

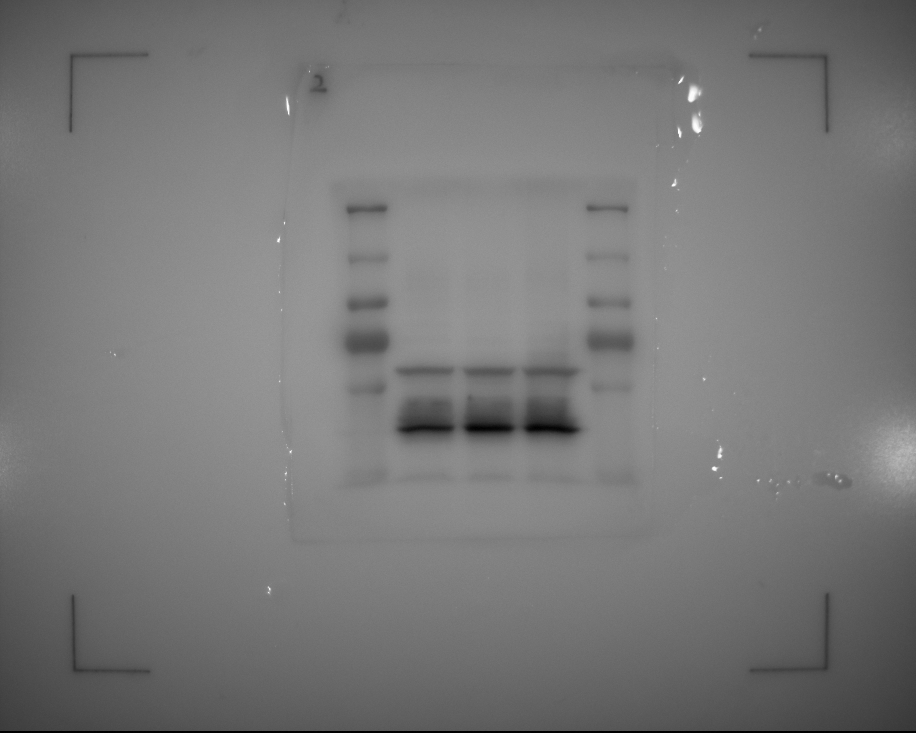

Supplement: Supplementary file 2 — Supplementary Material 2: CK19 [file 12964_2024_1656_MOESM2_ESM.zip › CK19/CK19_2 -20240130_192628_00.02_8bit.tif]

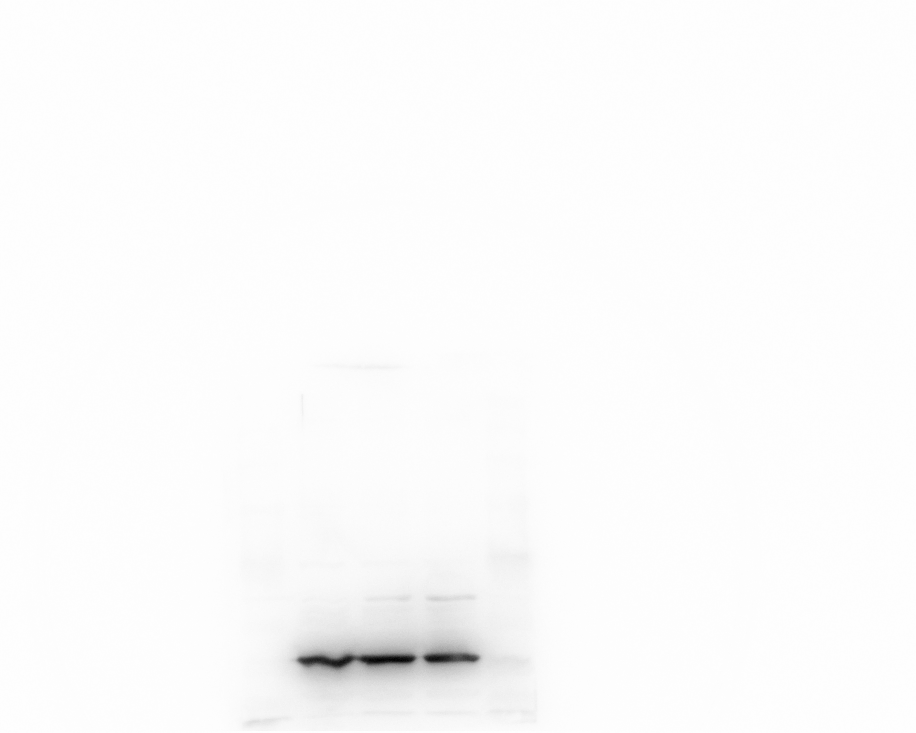

Supplement: Supplementary file 2 — Supplementary Material 2: CK19 [file 12964_2024_1656_MOESM2_ESM.zip › CK19/CK19_3_ 20240206_172925_00.00_8bit (1).tif]

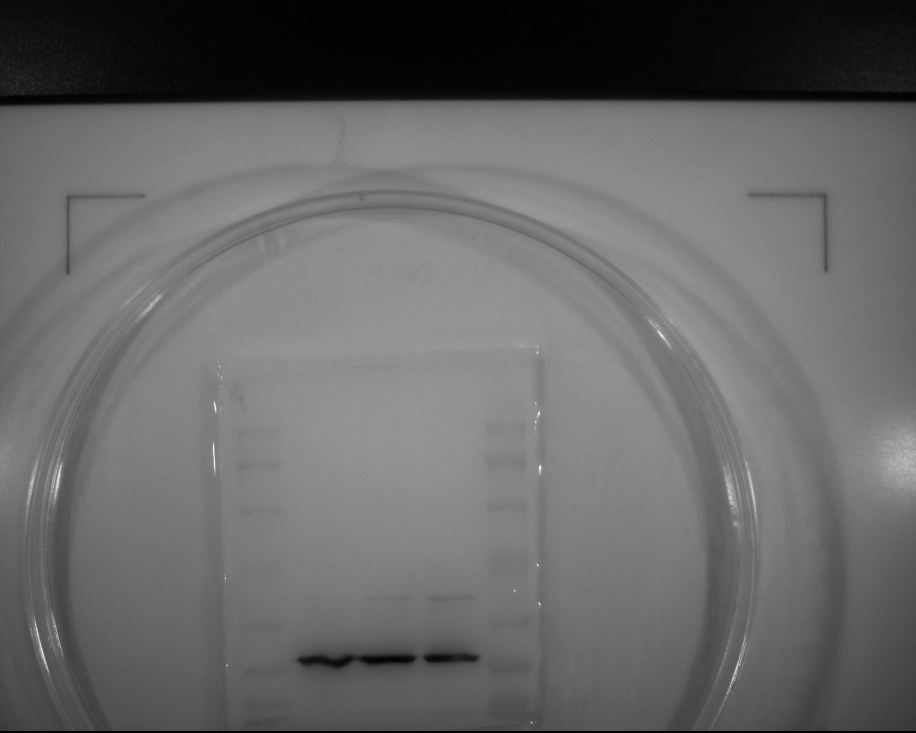

Supplement: Supplementary file 2 — Supplementary Material 2: CK19 [file 12964_2024_1656_MOESM2_ESM.zip › CK19/CK19_3_ 20240206_172925_00.00_8bit (2).tif]

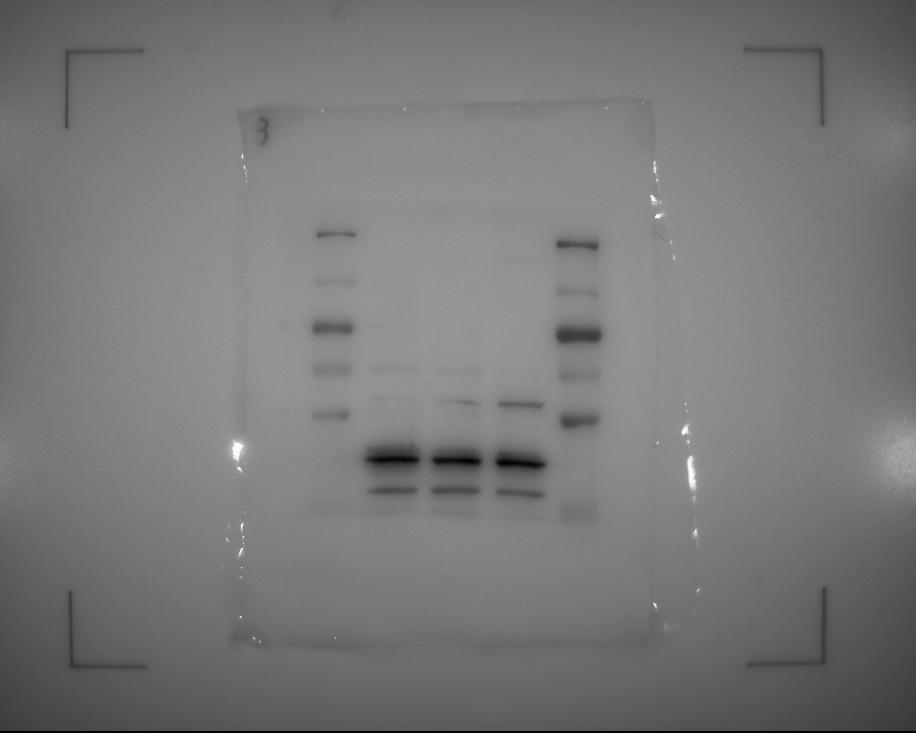

Supplement: Supplementary file 2 — Supplementary Material 2: CK19 [file 12964_2024_1656_MOESM2_ESM.zip › CK19/CK19-1 _20240201_164602_00.01_8bit(1).tif]

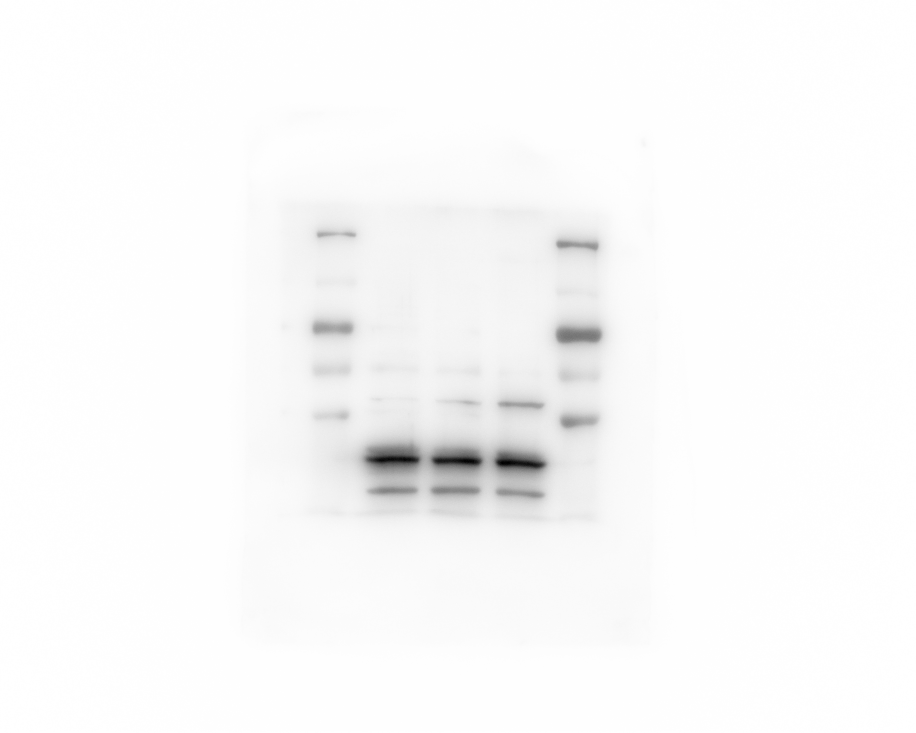

Supplement: Supplementary file 2 — Supplementary Material 2: CK19 [file 12964_2024_1656_MOESM2_ESM.zip › CK19/CK19-1 _20240201_164602_00.01_8bit.tif]

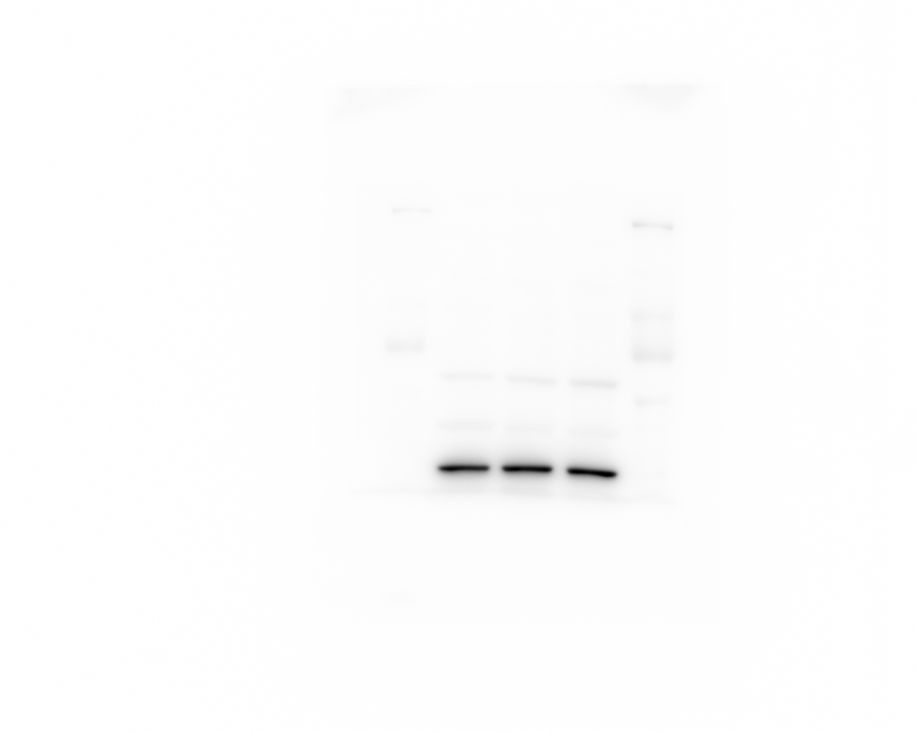

Supplement: Supplementary file 2 — Supplementary Material 2: CK19 [file 12964_2024_1656_MOESM2_ESM.zip › CK19/GAPDH_1 20240129_182800_00.02_8bit.tif]

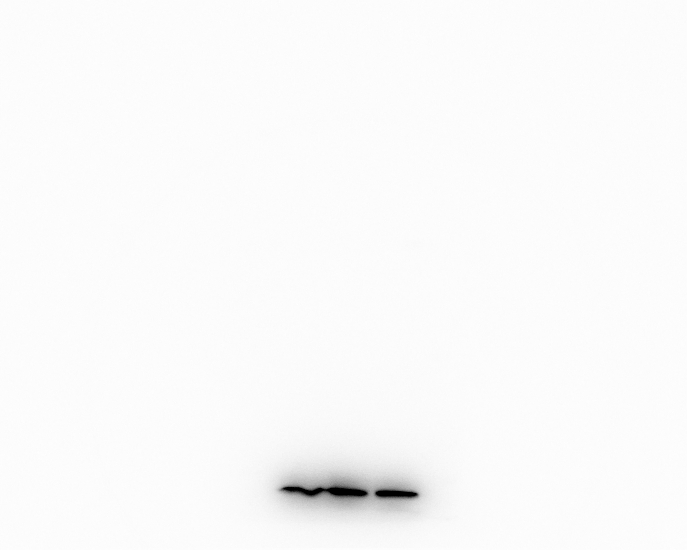

Supplement: Supplementary file 2 — Supplementary Material 2: CK19 [file 12964_2024_1656_MOESM2_ESM.zip › CK19/GAPDH_3 _20240204_213027_00.00_8bit (1).tif]

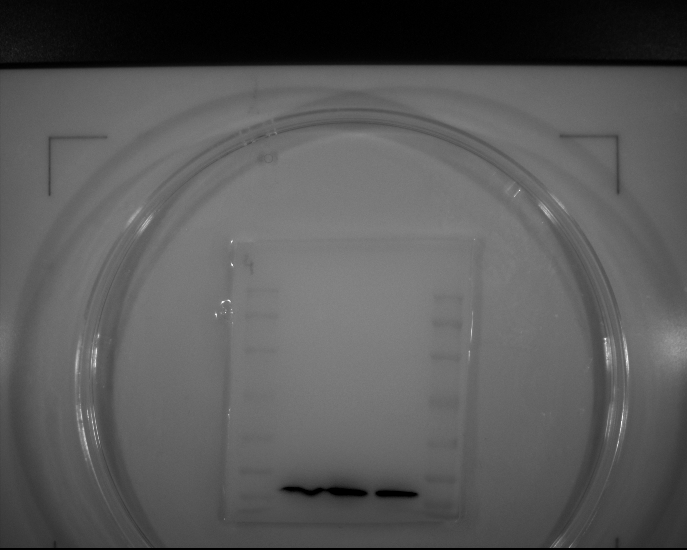

Supplement: Supplementary file 2 — Supplementary Material 2: CK19 [file 12964_2024_1656_MOESM2_ESM.zip › CK19/GAPDH_3 _20240204_213027_00.00_8bit (2).tif]

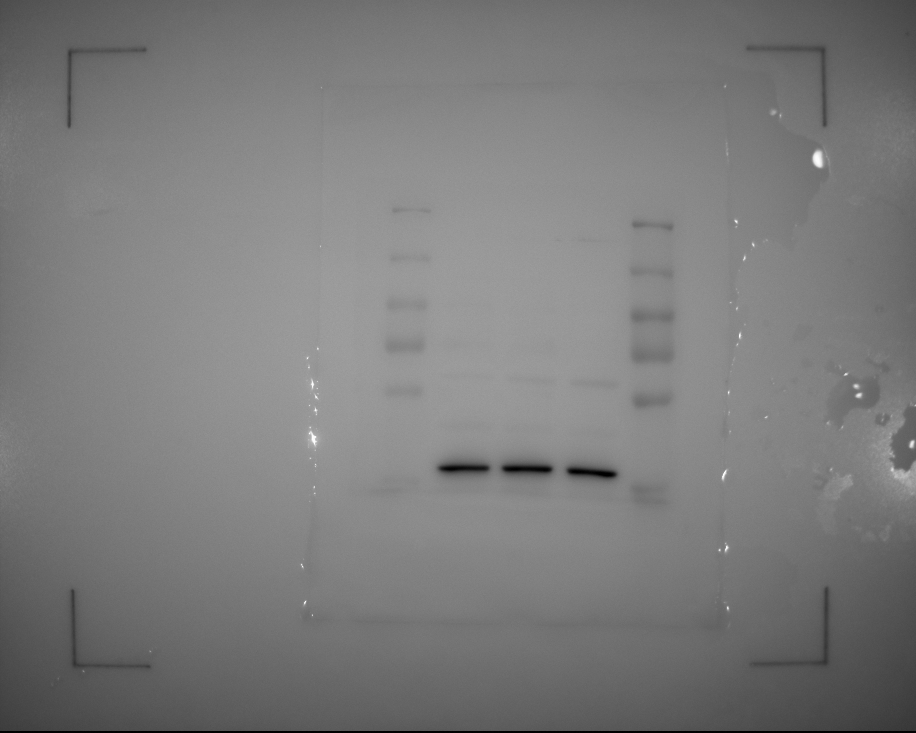

Supplement: Supplementary file 2 — Supplementary Material 2: CK19 [file 12964_2024_1656_MOESM2_ESM.zip › CK19/GAPDH-1 _20240129_182800_00.02_8bit(1).tif]

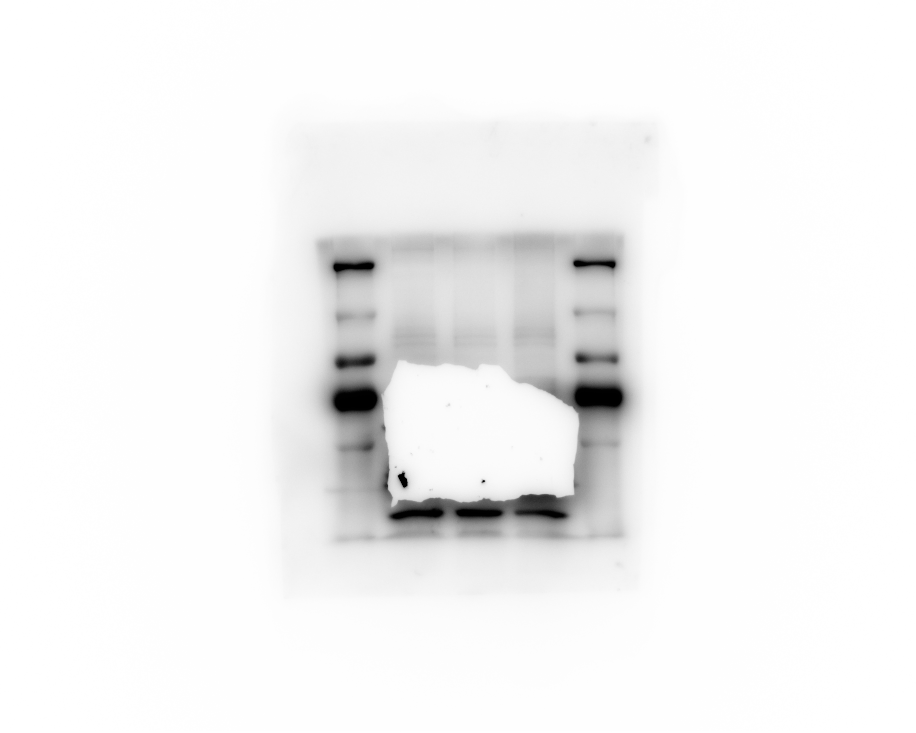

Supplement: Supplementary file 2 — Supplementary Material 2: CK19 [file 12964_2024_1656_MOESM2_ESM.zip › CK19/GAPDH-2_20240131_151448_00.03_8bit(0 (2).tif]

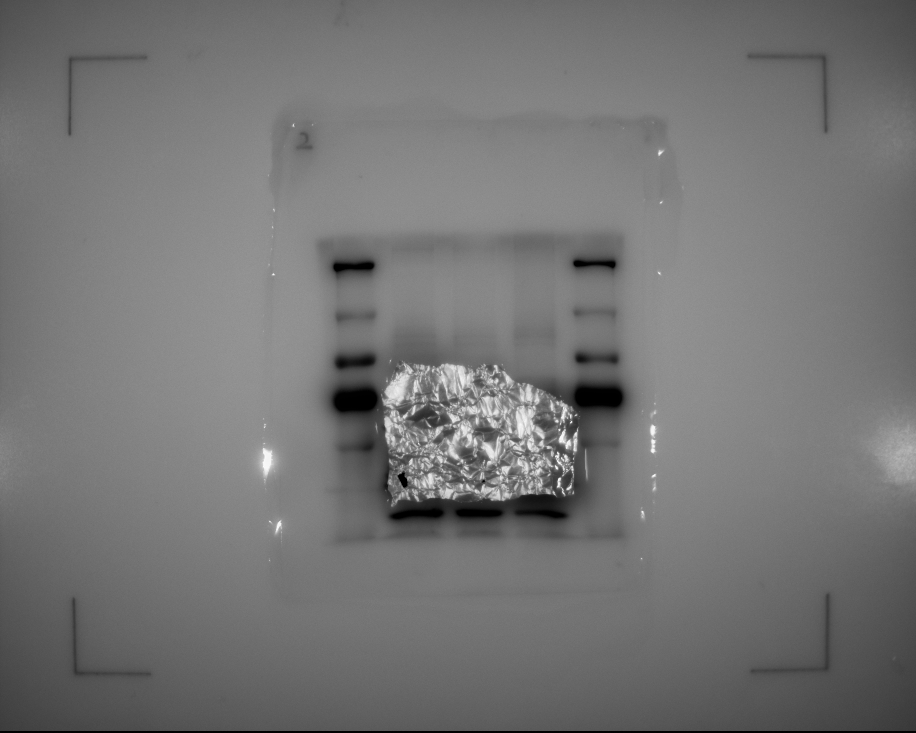

Supplement: Supplementary file 2 — Supplementary Material 2: CK19 [file 12964_2024_1656_MOESM2_ESM.zip › CK19/GAPDH-2_20240131_151448_00.03_8bit(0).tif]

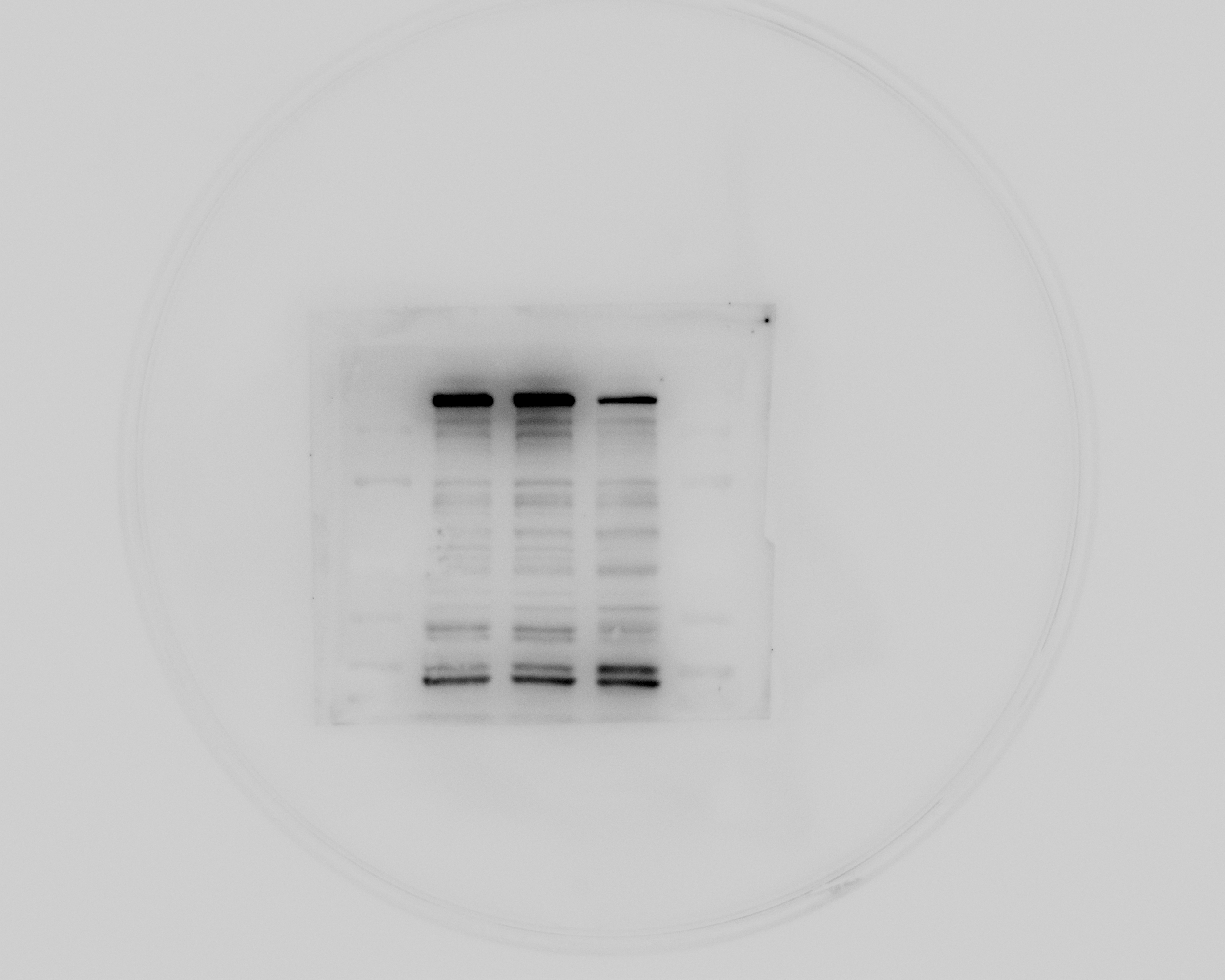

Supplement: Supplementary file 3 — Supplementary Material 3: Collagen I [file 12964_2024_1656_MOESM3_ESM.zip › Collagen I/COLLA_2_20240226_184449_01.37_8bit (1).tif]

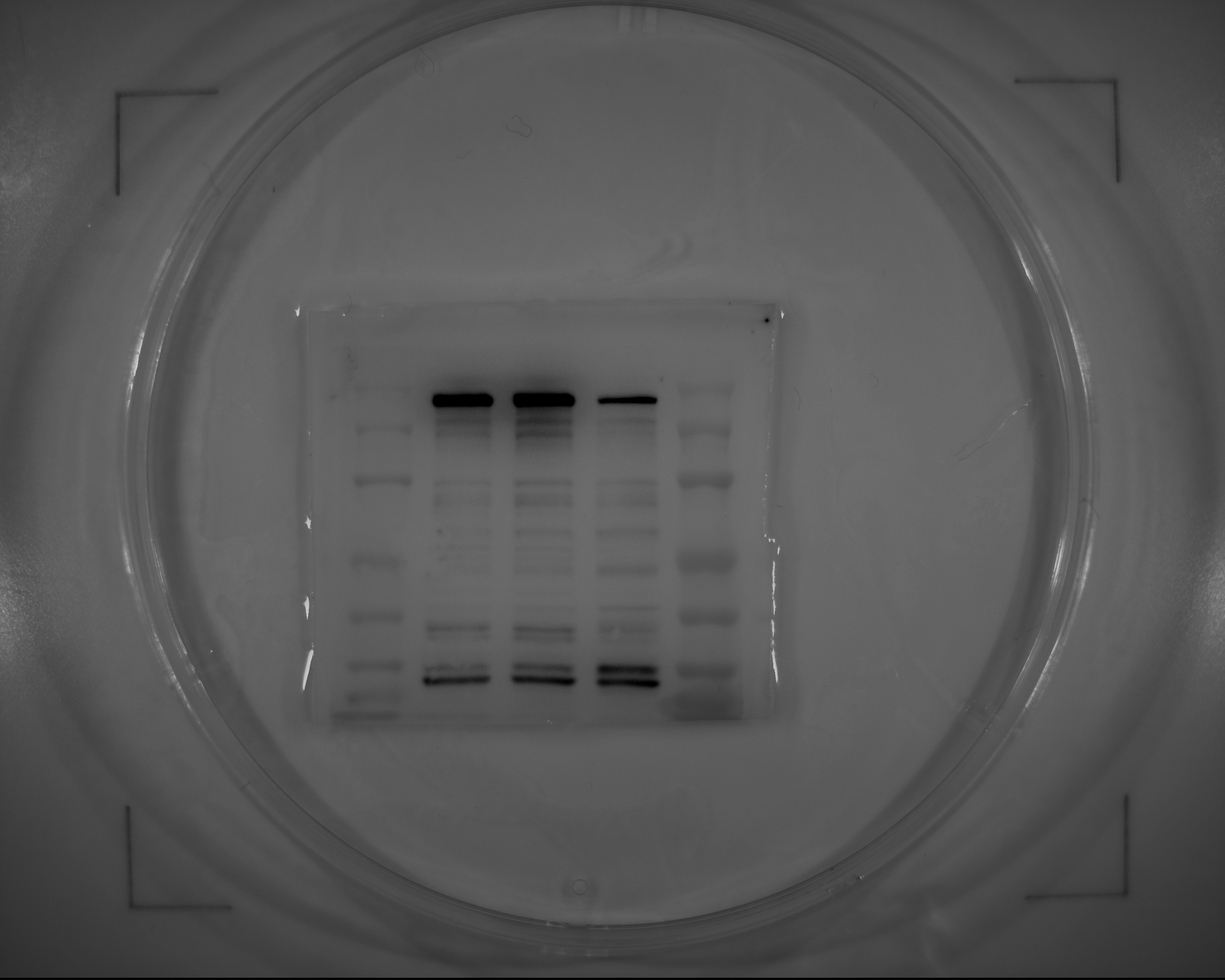

Supplement: Supplementary file 3 — Supplementary Material 3: Collagen I [file 12964_2024_1656_MOESM3_ESM.zip › Collagen I/COLLA_2_20240226_184449_01.37_8bit (2).tif]

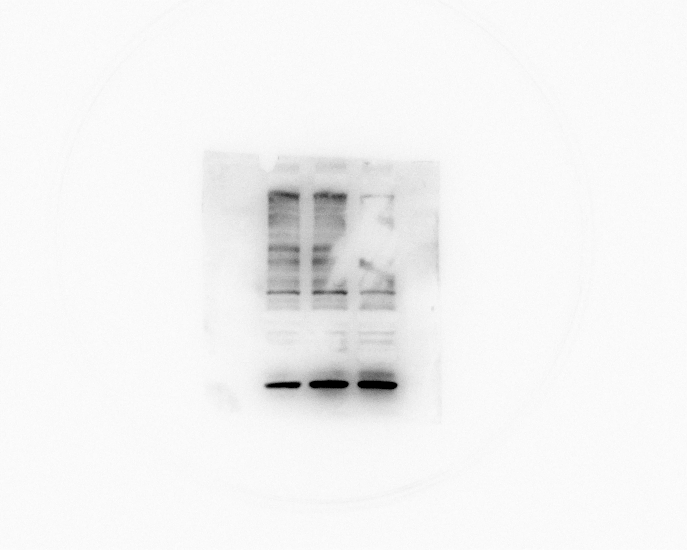

Supplement: Supplementary file 3 — Supplementary Material 3: Collagen I [file 12964_2024_1656_MOESM3_ESM.zip › Collagen I/COLLA_3_20240227_155231_00.04_8bit(0).tif]

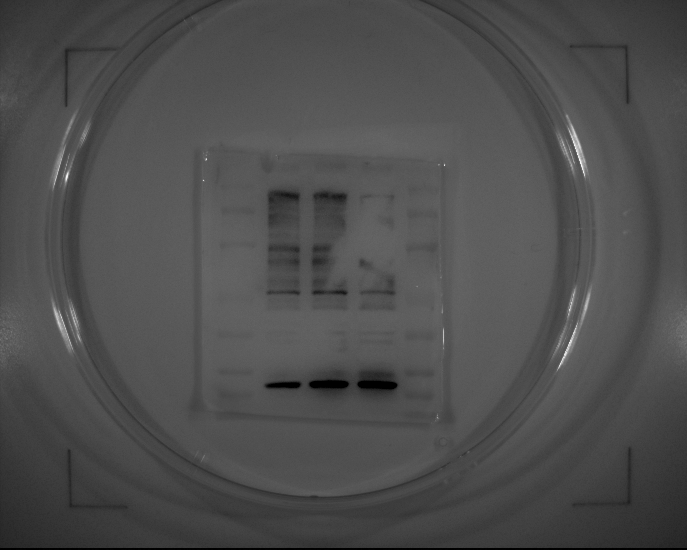

Supplement: Supplementary file 3 — Supplementary Material 3: Collagen I [file 12964_2024_1656_MOESM3_ESM.zip › Collagen I/COLLA_3_20240227_155231_00.04_8bit(1).tif]

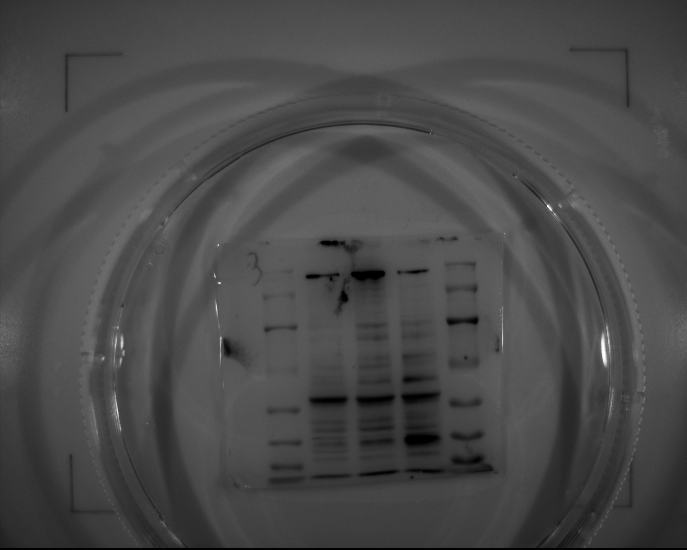

Supplement: Supplementary file 3 — Supplementary Material 3: Collagen I [file 12964_2024_1656_MOESM3_ESM.zip › Collagen I/COLLA-1_20240204_111448_01.00_8bit(0).tif]

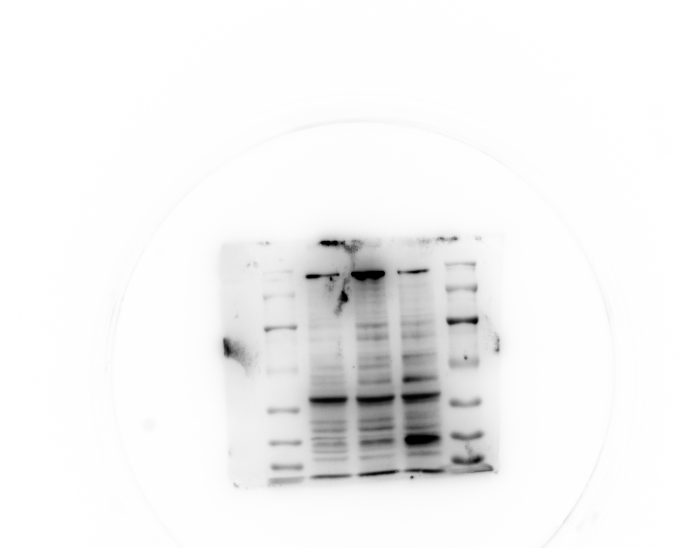

Supplement: Supplementary file 3 — Supplementary Material 3: Collagen I [file 12964_2024_1656_MOESM3_ESM.zip › Collagen I/COLLA-1_20240204_111448_01.00_8bit(1).tif]

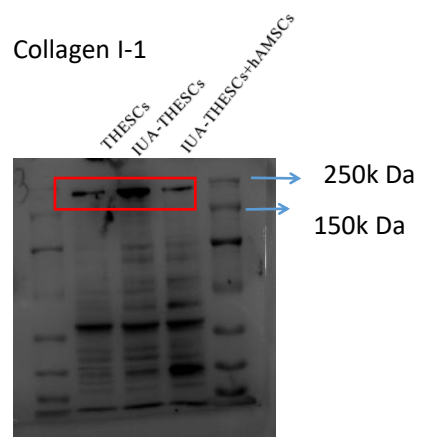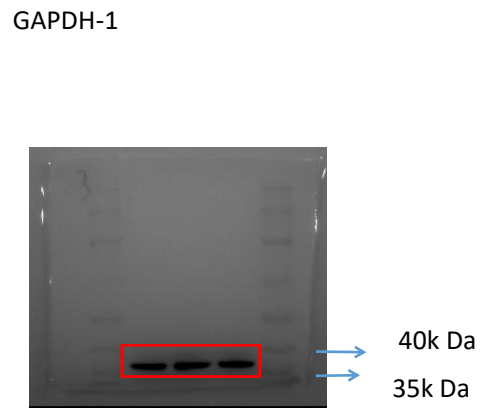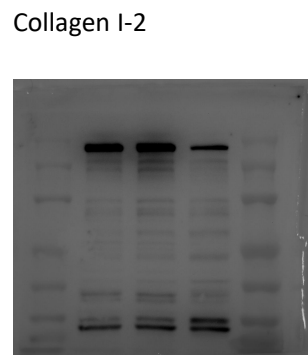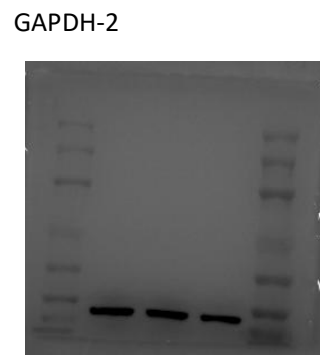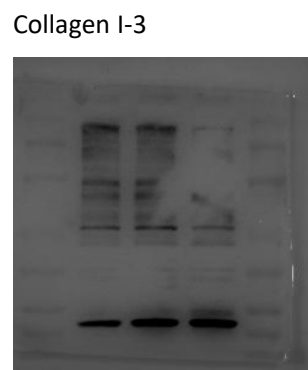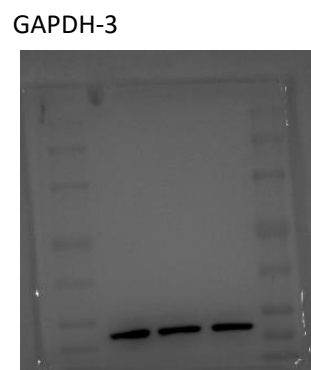

Supplement: Supplementary file 3 — Supplementary Material 3: Collagen I [file 12964_2024_1656_MOESM3_ESM.zip › Collagen I/CollagenI.pdf]

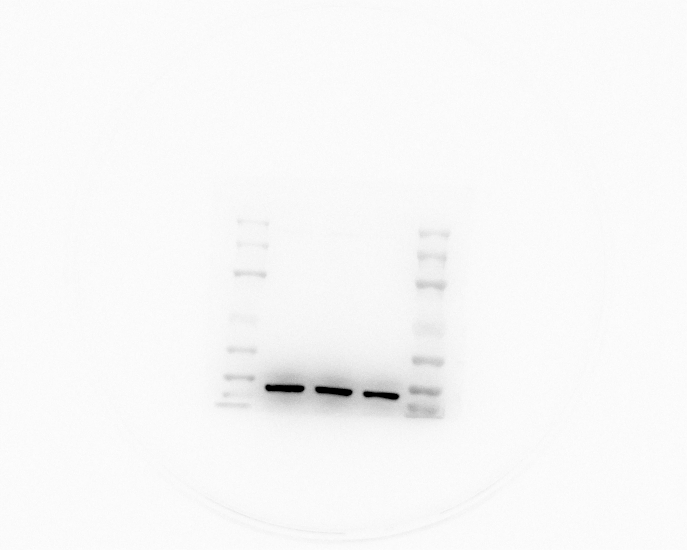

Supplement: Supplementary file 3 — Supplementary Material 3: Collagen I [file 12964_2024_1656_MOESM3_ESM.zip › Collagen I/GAPDH_2_20240227_215705_00.01_8bit (1).tif]

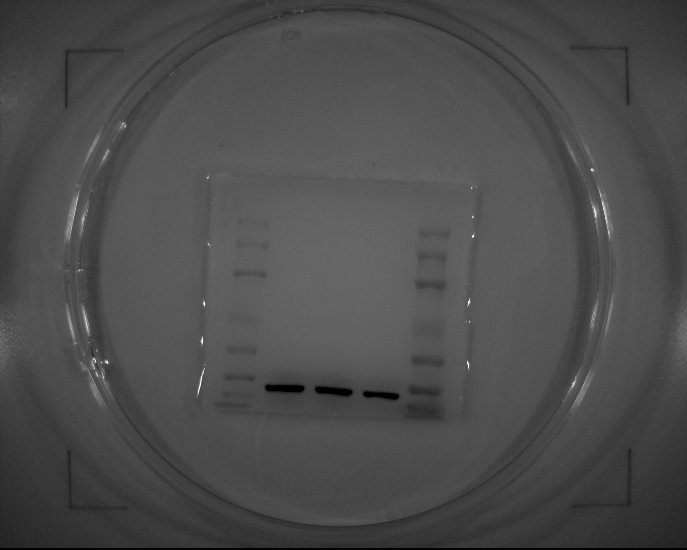

Supplement: Supplementary file 3 — Supplementary Material 3: Collagen I [file 12964_2024_1656_MOESM3_ESM.zip › Collagen I/GAPDH_2_20240227_215705_00.01_8bit (2).tif]

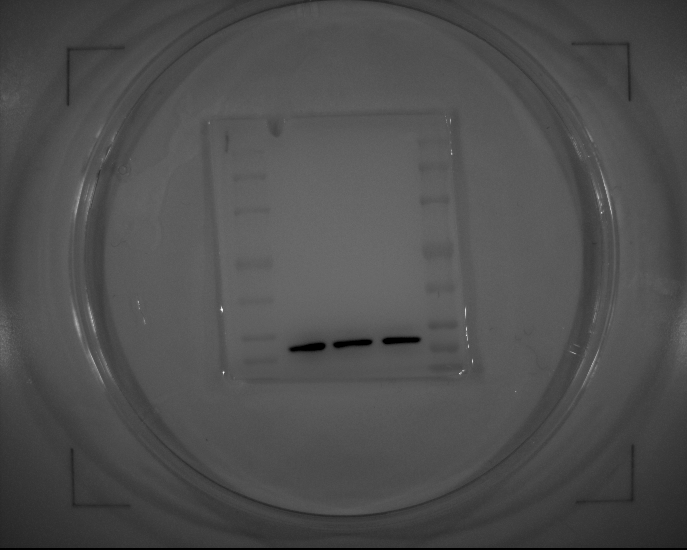

Supplement: Supplementary file 3 — Supplementary Material 3: Collagen I [file 12964_2024_1656_MOESM3_ESM.zip › Collagen I/GAPDH_3_20240225_203934_00.00_8bit (1).tif]

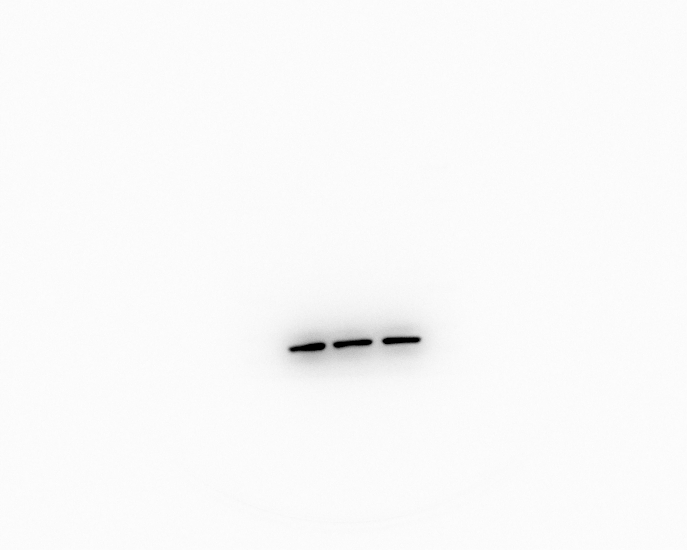

Supplement: Supplementary file 3 — Supplementary Material 3: Collagen I [file 12964_2024_1656_MOESM3_ESM.zip › Collagen I/GAPDH_3_20240225_203934_00.00_8bit (2).tif]

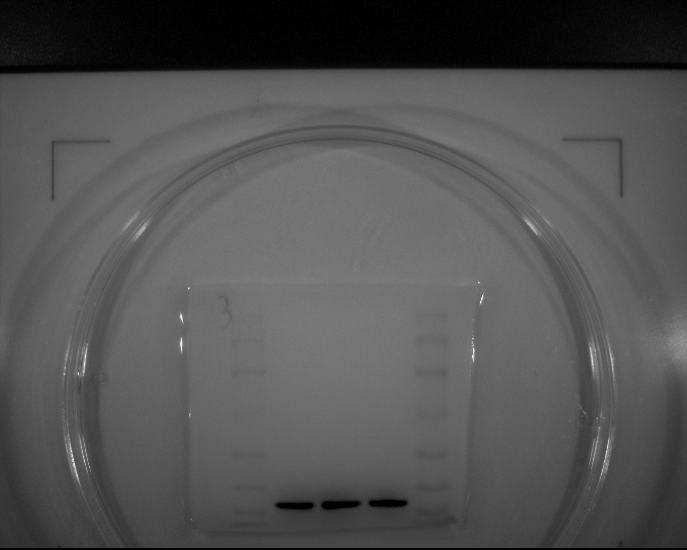

Supplement: Supplementary file 3 — Supplementary Material 3: Collagen I [file 12964_2024_1656_MOESM3_ESM.zip › Collagen I/GAPDH-1_2_20240204_212713_00.00_8bit(0).tif]

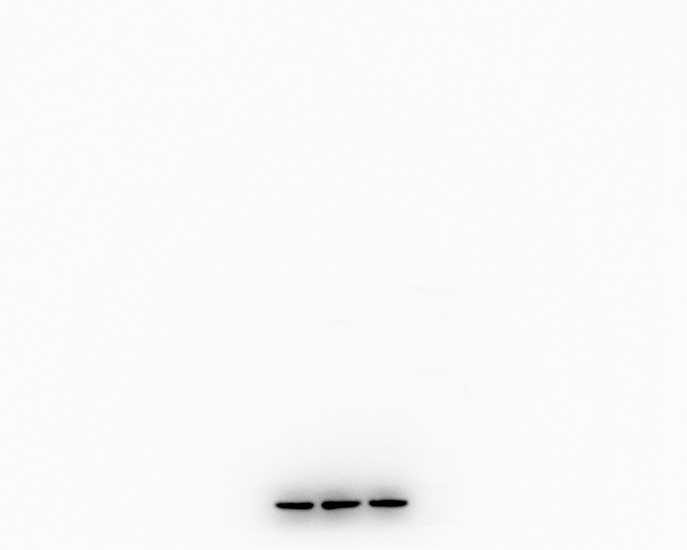

Supplement: Supplementary file 3 — Supplementary Material 3: Collagen I [file 12964_2024_1656_MOESM3_ESM.zip › Collagen I/GAPDH-1_2_20240204_212713_00.00_8bit(1).tif]

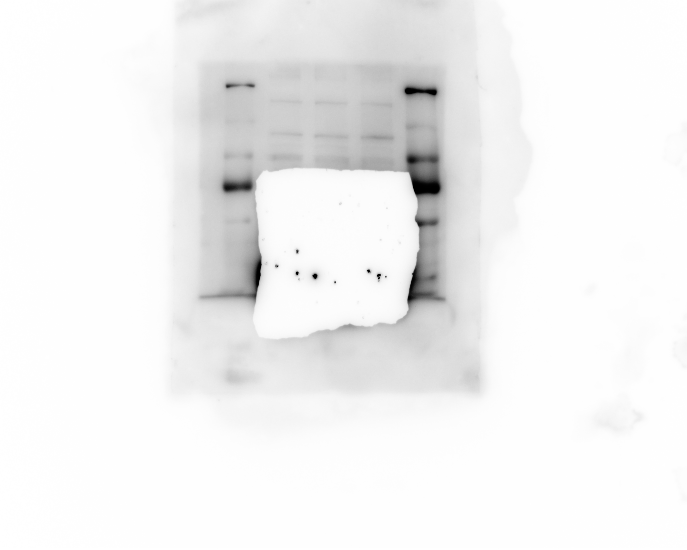

Supplement: Supplementary file 4 — Supplementary Material 4: E-cad [file 12964_2024_1656_MOESM4_ESM.zip › E-cad/E_cad_1 20240129_191620_03.00_8(1)bit.tif]

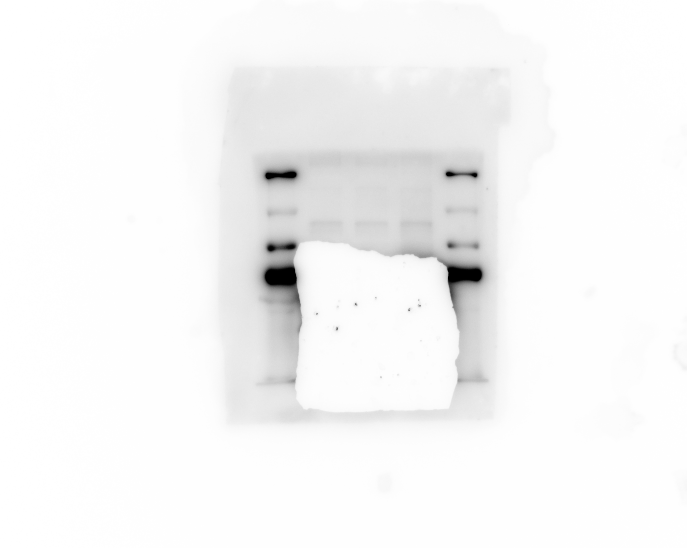

Supplement: Supplementary file 4 — Supplementary Material 4: E-cad [file 12964_2024_1656_MOESM4_ESM.zip › E-cad/E_cad_2 20240129_192234_03.00_8bit(2).tif]

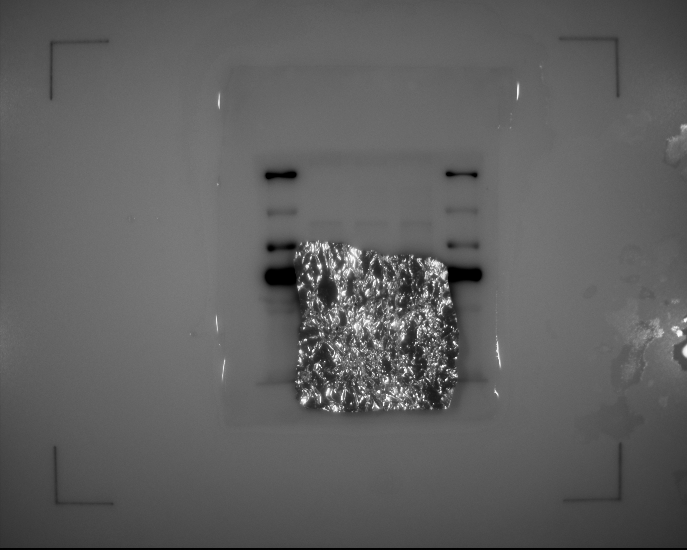

Supplement: Supplementary file 4 — Supplementary Material 4: E-cad [file 12964_2024_1656_MOESM4_ESM.zip › E-cad/E_cad_2 20240129_192234_03.00_8bit.tif]

E-cad-1

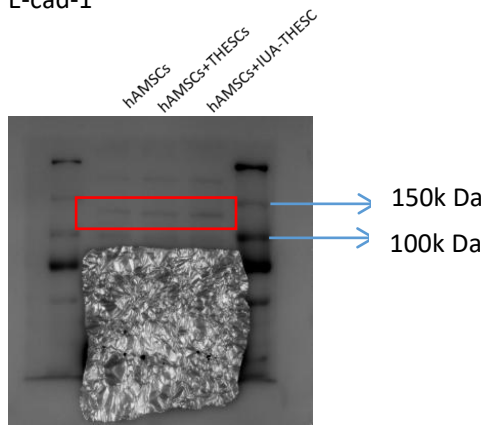

GAPDH-1

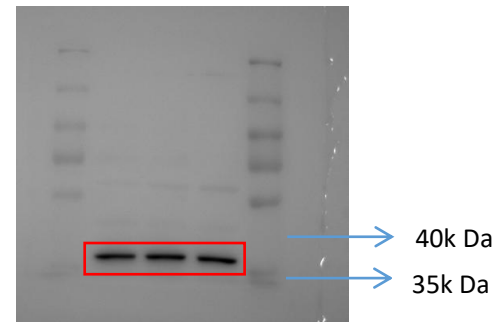

E-cad-2

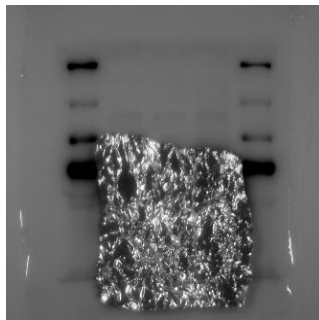

GAPDH-2

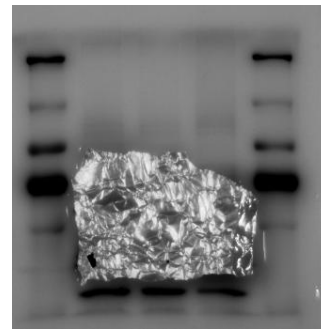

E-cad-3

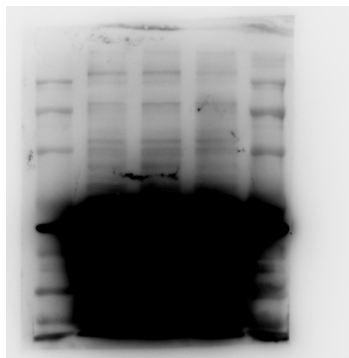

GAPDH-3

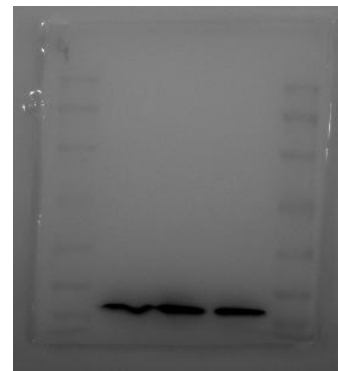

Supplement: Supplementary file 4 — Supplementary Material 4: E-cad [file 12964_2024_1656_MOESM4_ESM.zip › E-cad/E-cad.pdf]

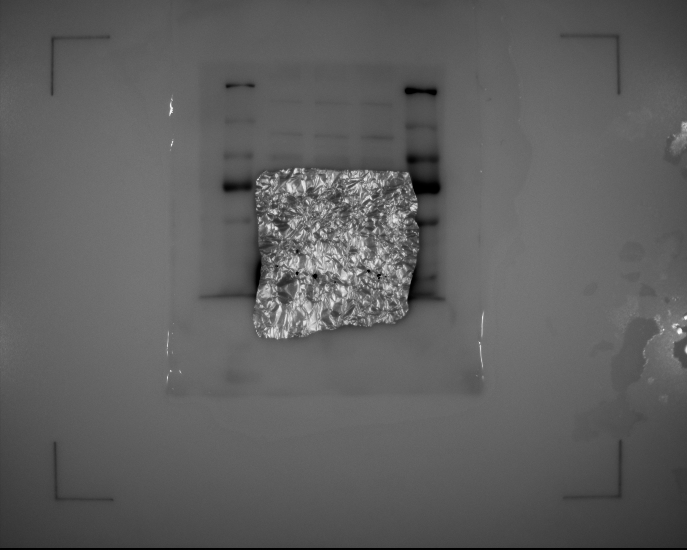

Supplement: Supplementary file 4 — Supplementary Material 4: E-cad [file 12964_2024_1656_MOESM4_ESM.zip › E-cad/E-cad-1 20240129_191620_03.00_8bit.tif]

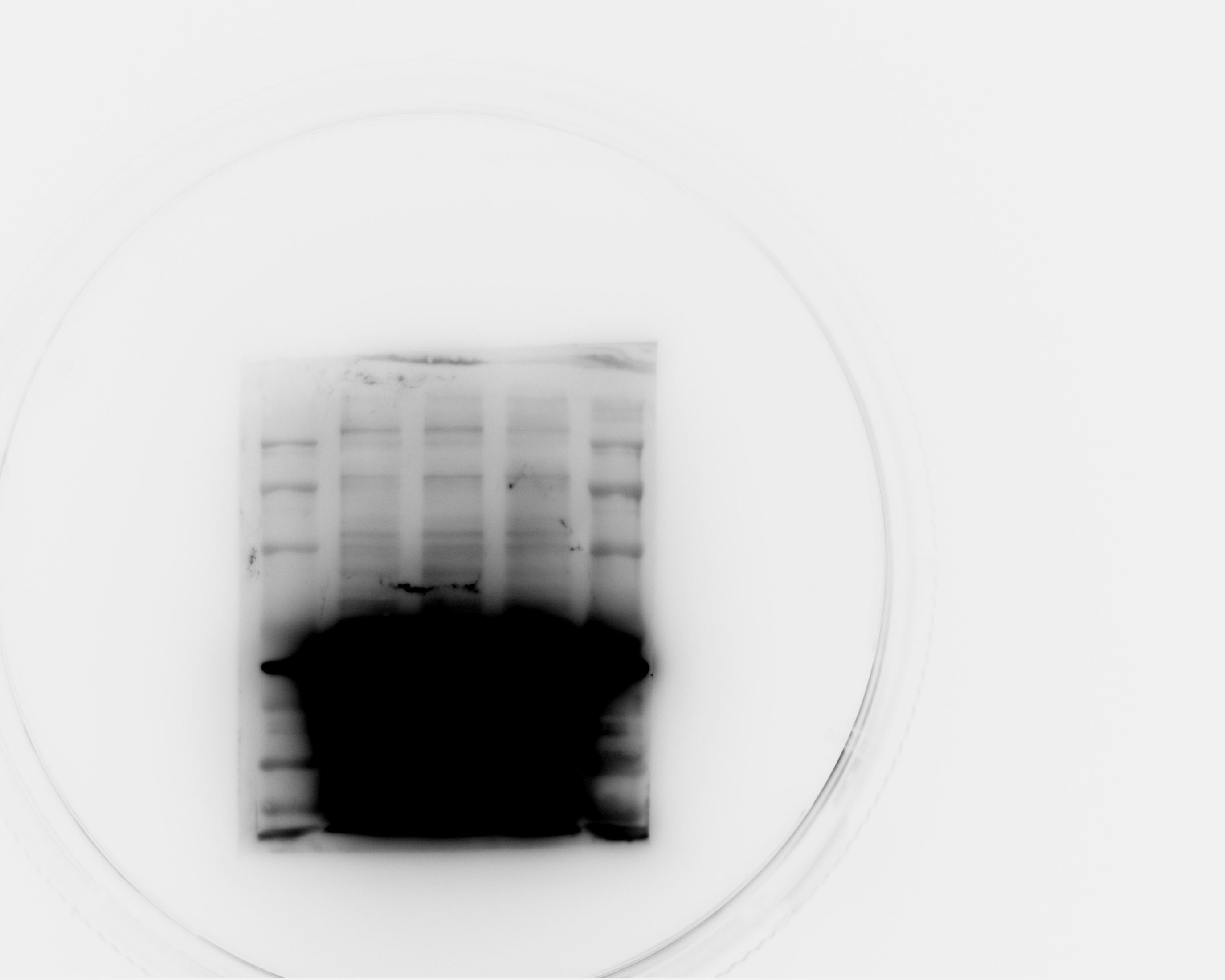

Supplement: Supplementary file 4 — Supplementary Material 4: E-cad [file 12964_2024_1656_MOESM4_ESM.zip › E-cad/E-cad-3_ 20240204_112040_01.00_8bit(0).tif]

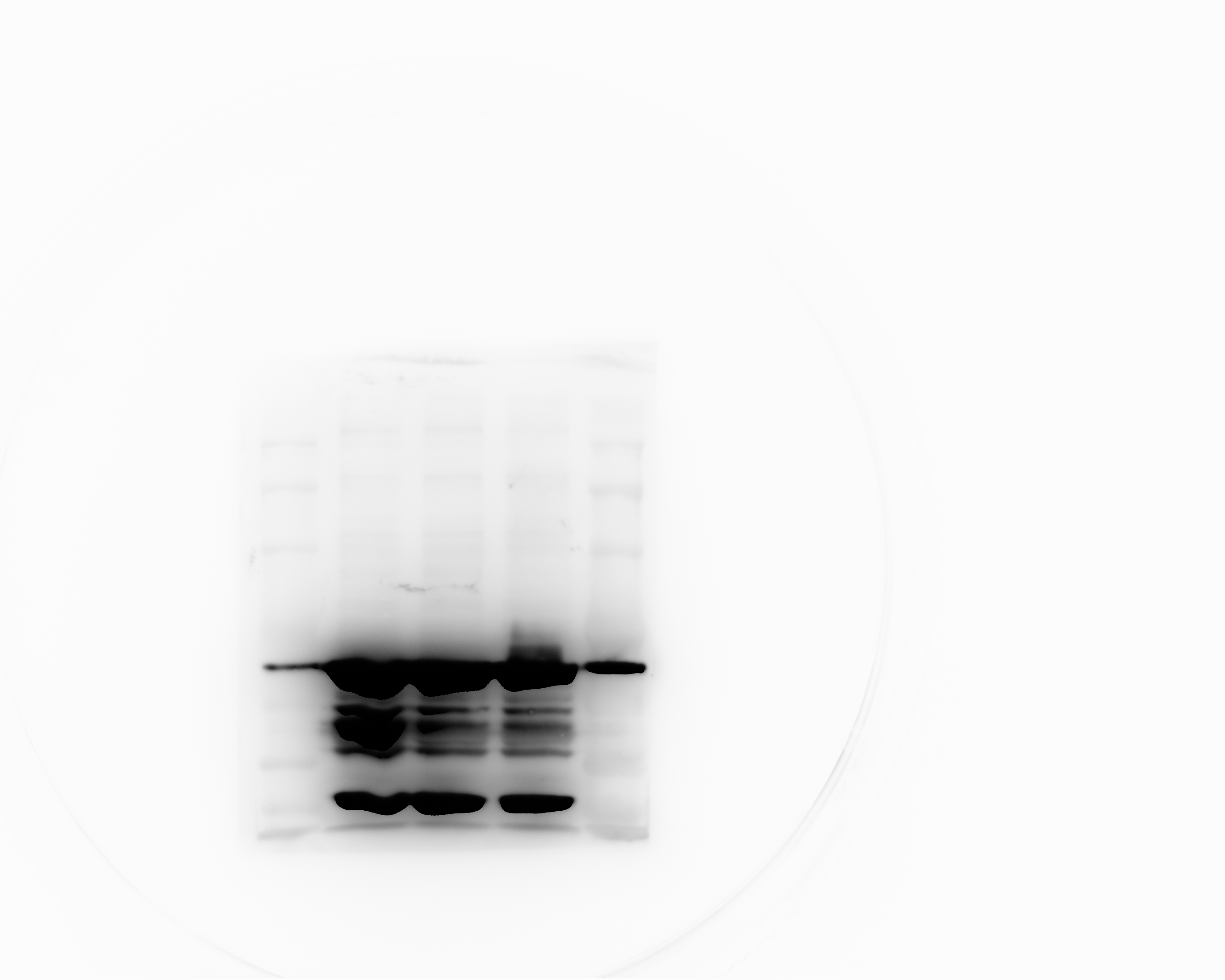

Supplement: Supplementary file 4 — Supplementary Material 4: E-cad [file 12964_2024_1656_MOESM4_ESM.zip › E-cad/E-cad-3_ 20240204_112040_01.00_8bit(1).tif]

FN-1

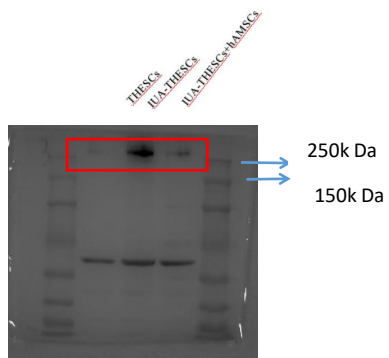

GAPDH-1

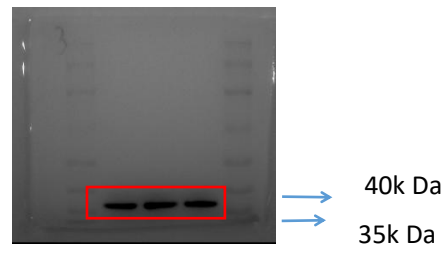

FN-2

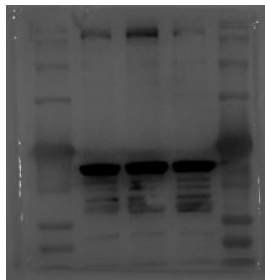

GAPDH-2

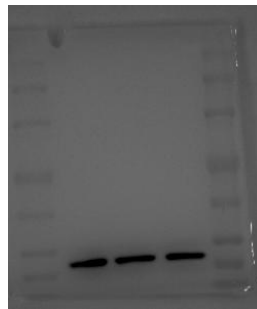

FN-3

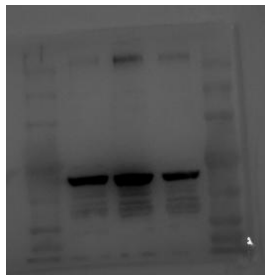

GAPDH-3

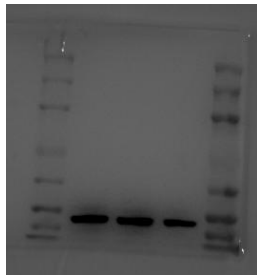

Supplement: Supplementary file 5 — Supplementary Material 5: FN [file 12964_2024_1656_MOESM5_ESM.zip › FN/FN.pdf]

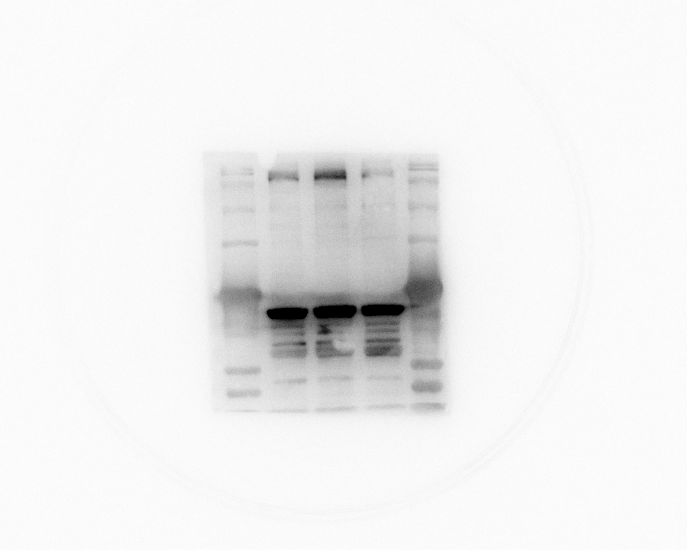

Supplement: Supplementary file 5 — Supplementary Material 5: FN [file 12964_2024_1656_MOESM5_ESM.zip › FN/FN_2_20240226_182046_00.00_8bit (1).tif]

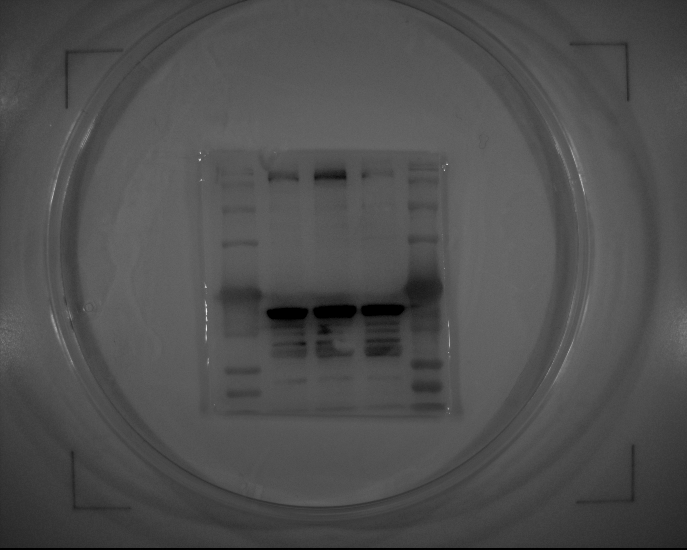

Supplement: Supplementary file 5 — Supplementary Material 5: FN [file 12964_2024_1656_MOESM5_ESM.zip › FN/FN_2_20240226_182046_00.00_8bit (2).tif]

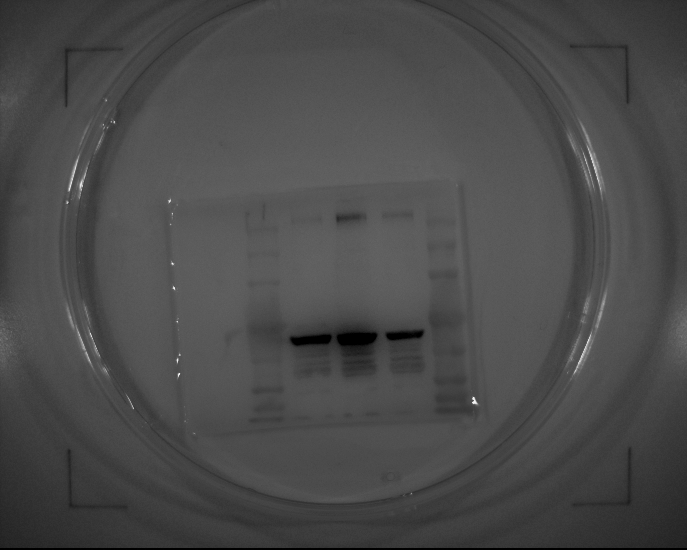

Supplement: Supplementary file 5 — Supplementary Material 5: FN [file 12964_2024_1656_MOESM5_ESM.zip › FN/FN_3_20240227_160107_00.00_8bit (1).tif]

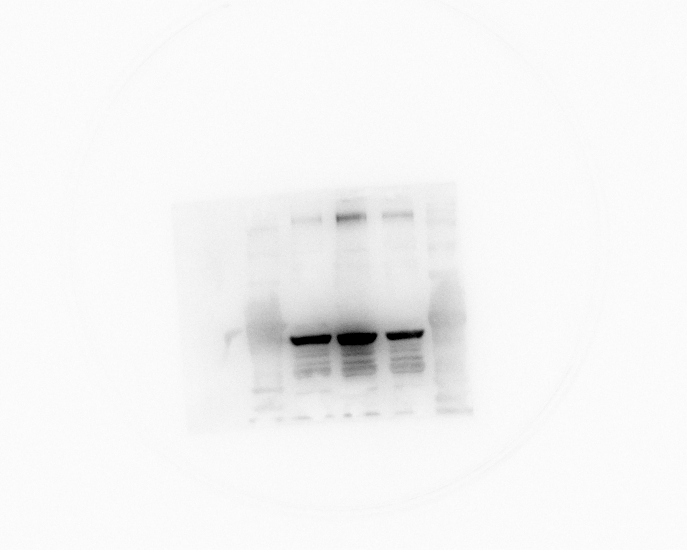

Supplement: Supplementary file 5 — Supplementary Material 5: FN [file 12964_2024_1656_MOESM5_ESM.zip › FN/FN_3_20240227_160107_00.00_8bit (2).tif]

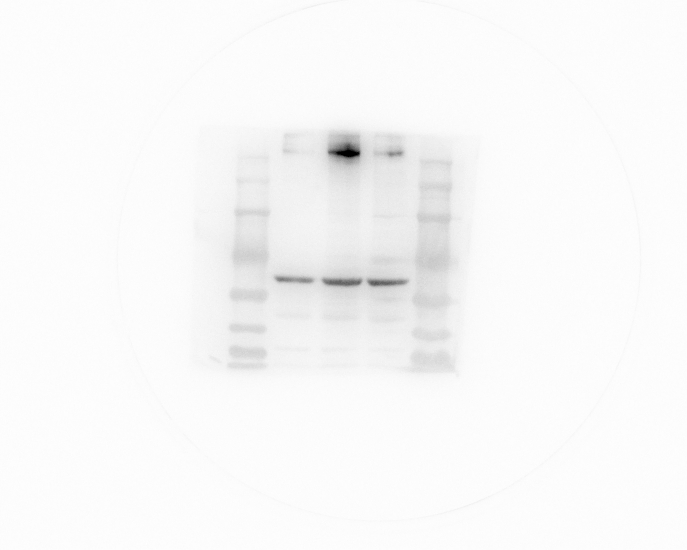

Supplement: Supplementary file 5 — Supplementary Material 5: FN [file 12964_2024_1656_MOESM5_ESM.zip › FN/FN-1_20240205_181055_00.00_8bit (1).tif]

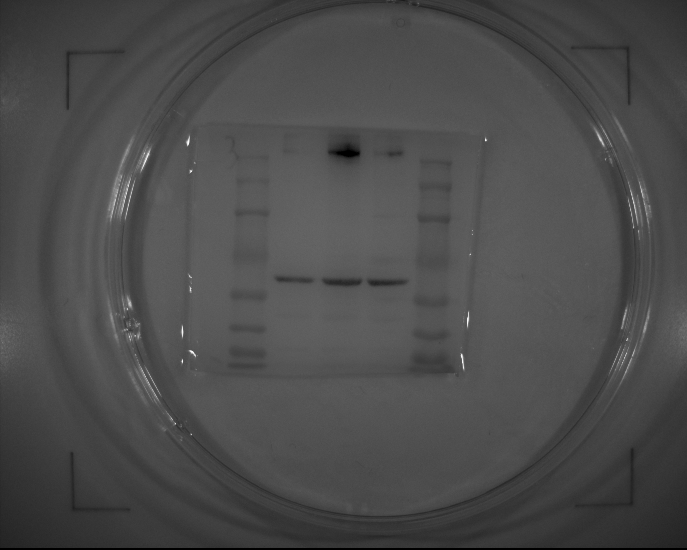

Supplement: Supplementary file 5 — Supplementary Material 5: FN [file 12964_2024_1656_MOESM5_ESM.zip › FN/FN-1_20240205_181055_00.00_8bit (2).tif]

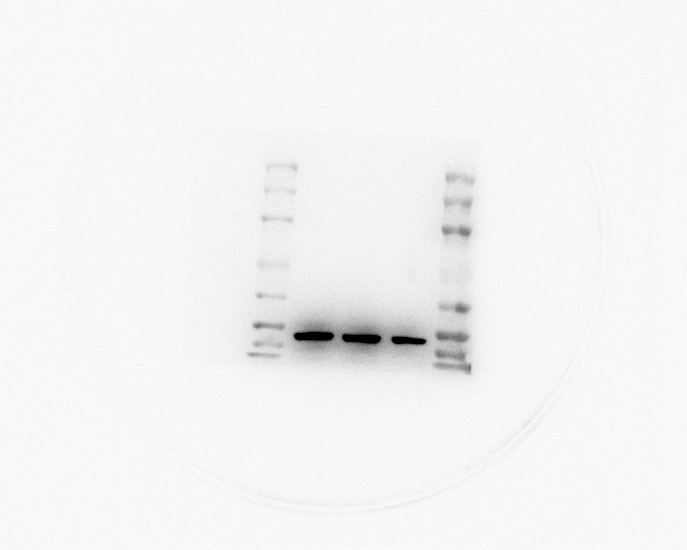

Supplement: Supplementary file 5 — Supplementary Material 5: FN [file 12964_2024_1656_MOESM5_ESM.zip › FN/GAPDH_3_20240227_215556_00.02_8bit (1).tif]

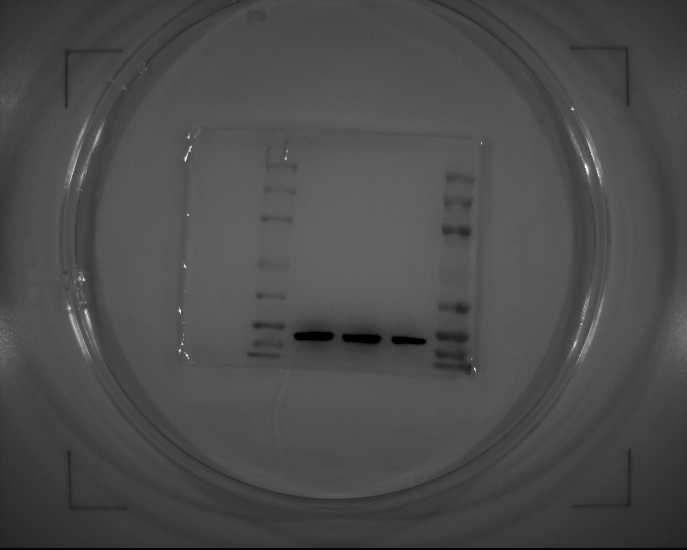

Supplement: Supplementary file 5 — Supplementary Material 5: FN [file 12964_2024_1656_MOESM5_ESM.zip › FN/GAPDH_3_20240227_215556_00.02_8bit (2).tif]

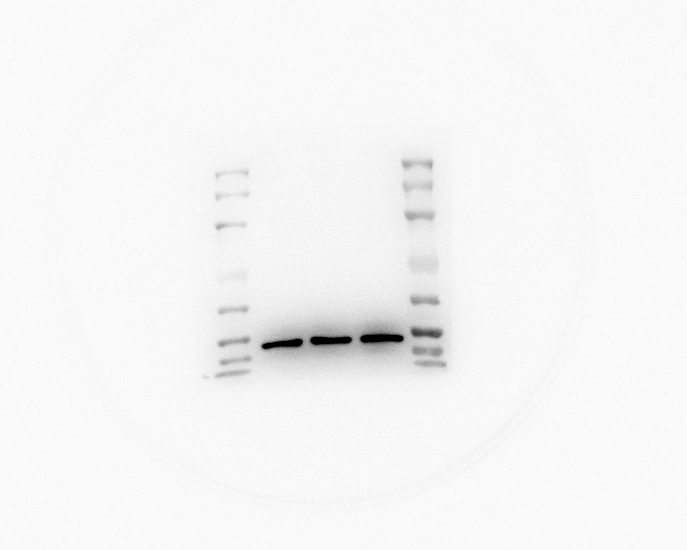

Supplement: Supplementary file 6 — Supplementary Material 6: Vimentin [file 12964_2024_1656_MOESM6_ESM.zip › Vimentin/GAPDH_2_20240227_215957_00.02_8bit (1).tif]

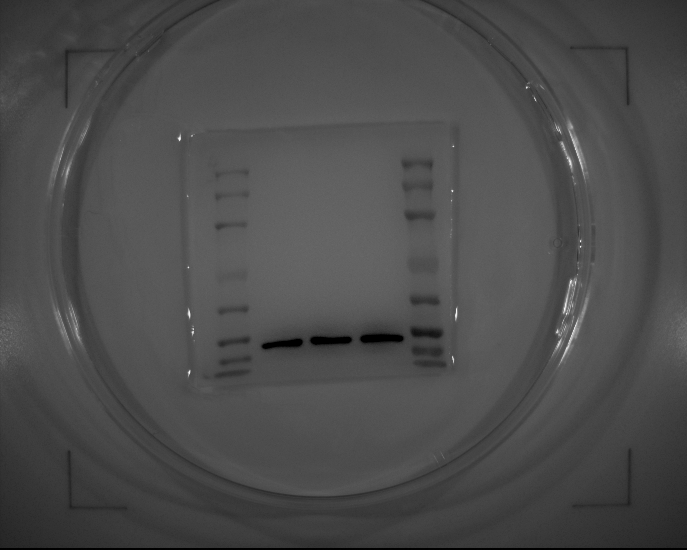

Supplement: Supplementary file 6 — Supplementary Material 6: Vimentin [file 12964_2024_1656_MOESM6_ESM.zip › Vimentin/GAPDH_2_20240227_215957_00.02_8bit (2).tif]

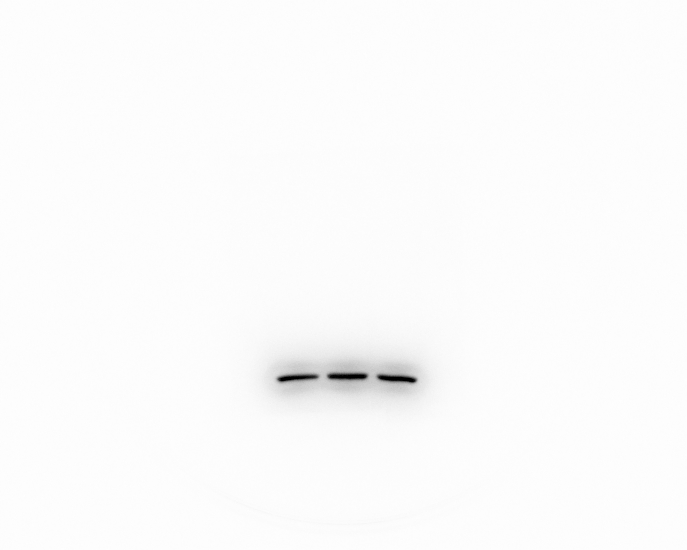

Supplement: Supplementary file 6 — Supplementary Material 6: Vimentin [file 12964_2024_1656_MOESM6_ESM.zip › Vimentin/GAPDH_3_20240225_204054_00.00_8bit (1).tif]

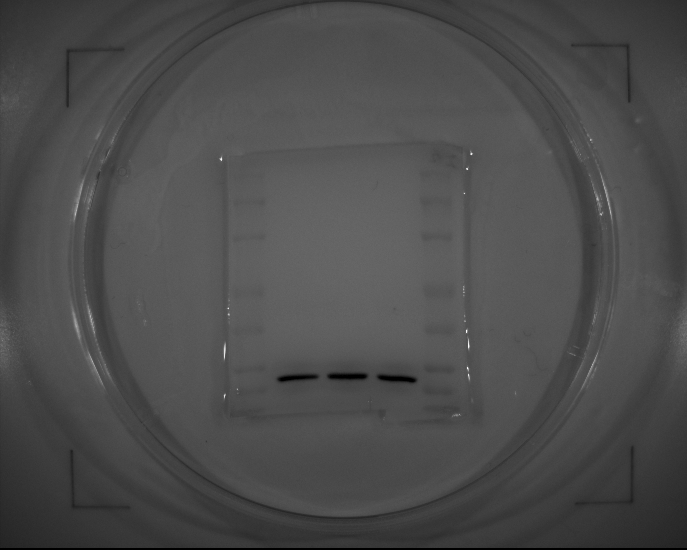

Supplement: Supplementary file 6 — Supplementary Material 6: Vimentin [file 12964_2024_1656_MOESM6_ESM.zip › Vimentin/GAPDH_3_20240225_204054_00.00_8bit (2).tif]

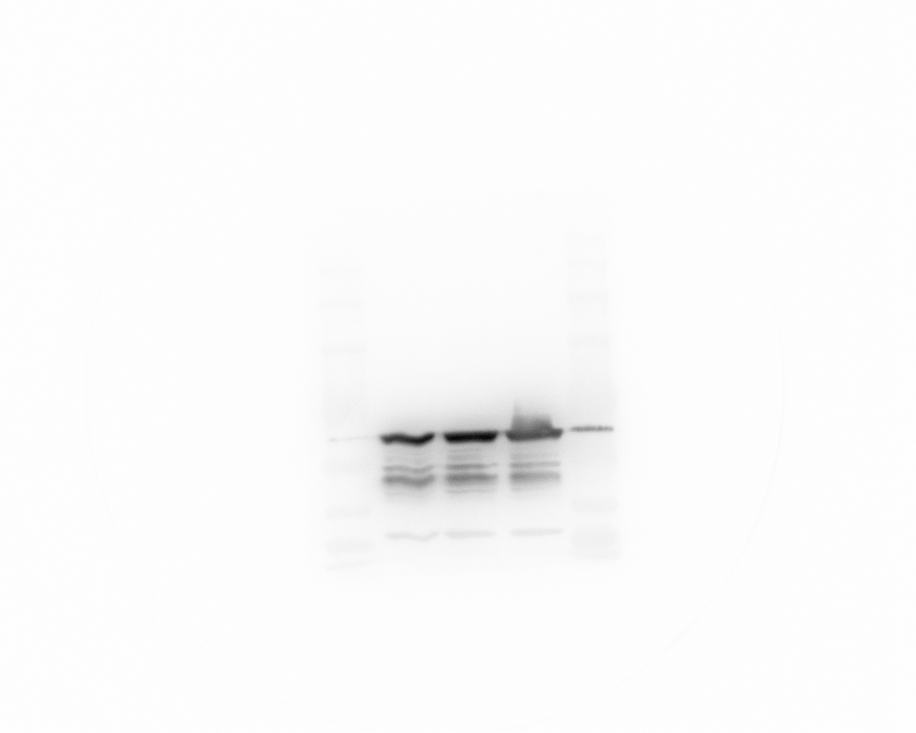

Supplement: Supplementary file 6 — Supplementary Material 6: Vimentin [file 12964_2024_1656_MOESM6_ESM.zip › Vimentin/VIM_1_H_20240205_181401_00.00_8bit(0).tif]

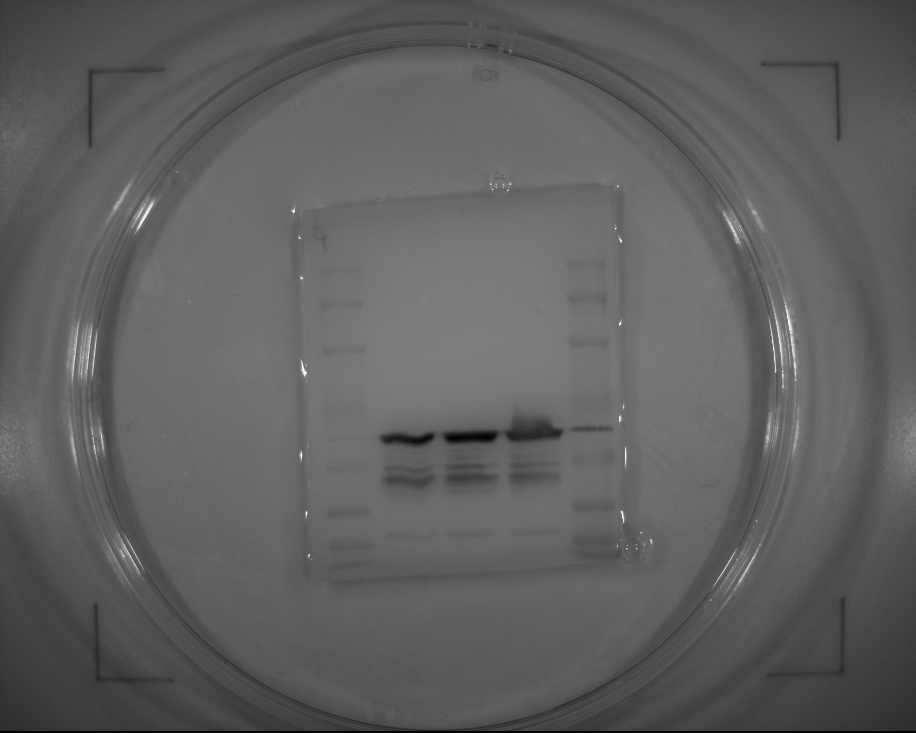

Supplement: Supplementary file 6 — Supplementary Material 6: Vimentin [file 12964_2024_1656_MOESM6_ESM.zip › Vimentin/VIM_1_H_20240205_181401_00.00_8bit(1).tif]

Vimentin-1

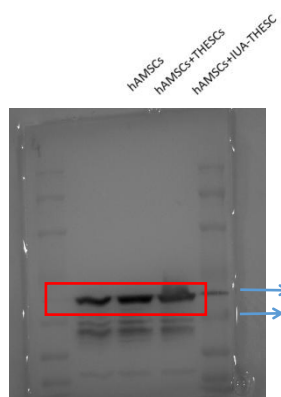

GAPDH-1

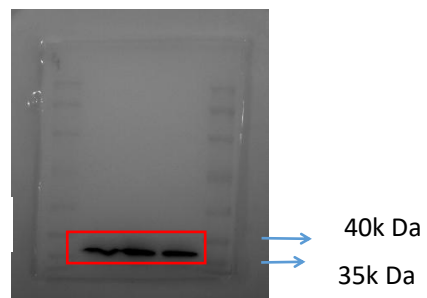

Vimentin-2

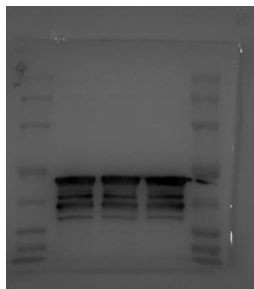

GAPDH-2

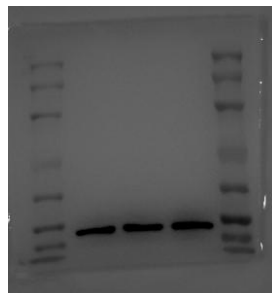

Vimentin-3

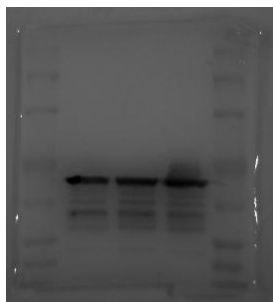

GAPDH-3

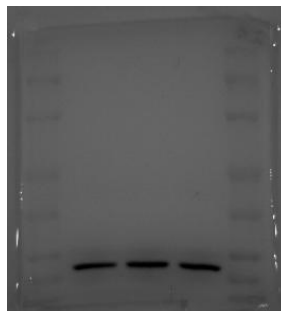

Supplement: Supplementary file 6 — Supplementary Material 6: Vimentin [file 12964_2024_1656_MOESM6_ESM.zip › Vimentin/Vimentin.pdf]

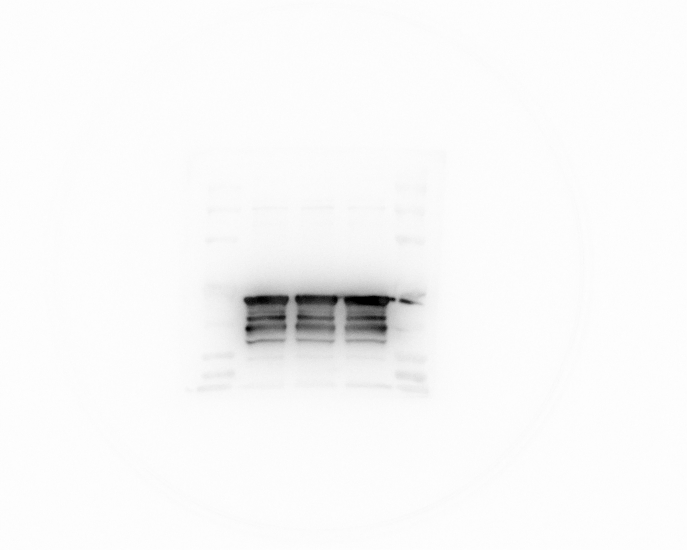

Supplement: Supplementary file 6 — Supplementary Material 6: Vimentin [file 12964_2024_1656_MOESM6_ESM.zip › Vimentin/VIMENTIN_2_20240226_185307_00.01_8bit (1).tif]

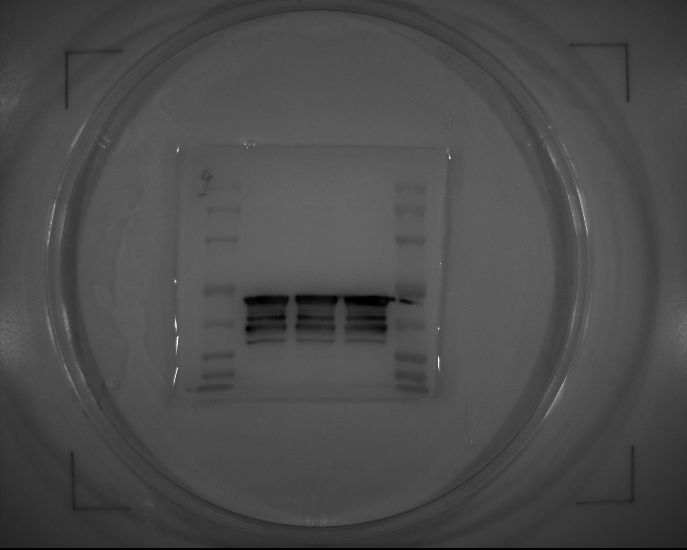

Supplement: Supplementary file 6 — Supplementary Material 6: Vimentin [file 12964_2024_1656_MOESM6_ESM.zip › Vimentin/VIMENTIN_2_20240226_185307_00.01_8bit (2).tif]

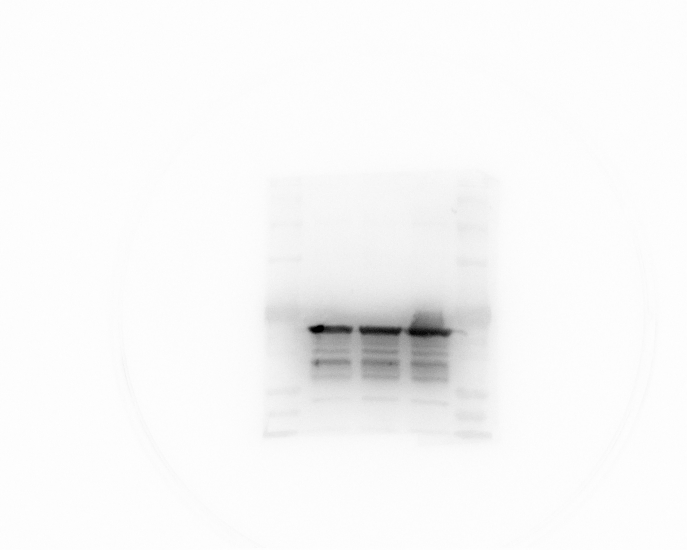

Supplement: Supplementary file 6 — Supplementary Material 6: Vimentin [file 12964_2024_1656_MOESM6_ESM.zip › Vimentin/VIMENTIN_3_20240226_182346_00.00_8bit (1).tif]

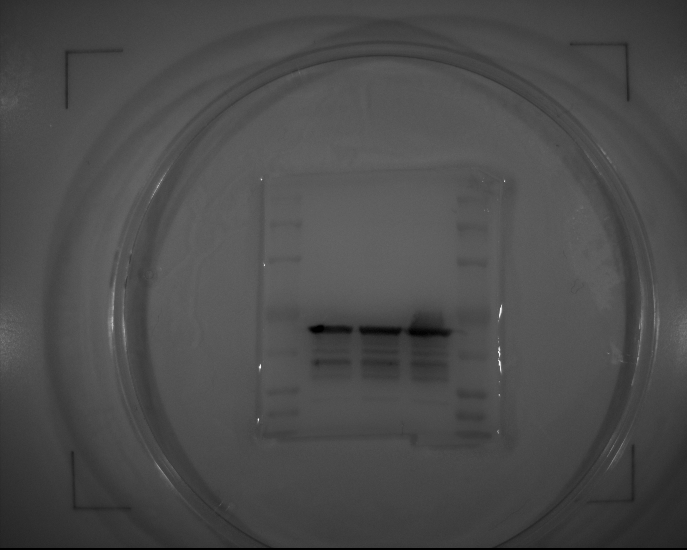

Supplement: Supplementary file 6 — Supplementary Material 6: Vimentin [file 12964_2024_1656_MOESM6_ESM.zip › Vimentin/VIMENTIN_3_20240226_182346_00.00_8bit (2).tif]
