# Supplementary material for: Human amnion mesenchymal stem cells promote endometrial repair via paracrine, preferentially than transdifferentiation
Source: Cell Commun Signal. 2024 May 31;22:301. doi: 10.1186/s12964-024-01656-0 (PMC11140932; doi:10.1186/s12964-024-01656-0)
Supplement: Supplementary file 2 — Supplementary Material 2: CK19 [file 12964_2024_1656_MOESM2_ESM.zip › CK19/CK19.doc]

CK19-1	GAPDH-1

55k Da

35k Da

55k Da

35k Da

CK19-2	GAPDH-2

CK19-3	GAPDH-3

40k Da

35k Da

40k Da

35k Da

（different marker）
