# Supplementary material for: Human amnion mesenchymal stem cells promote endometrial repair via paracrine, preferentially than transdifferentiation
Source: Cell Commun Signal. 2024 May 31;22:301. doi: 10.1186/s12964-024-01656-0 (PMC11140932; doi:10.1186/s12964-024-01656-0)
Supplement: Supplementary file 3 — Supplementary Material 3: Collagen I [file 12964_2024_1656_MOESM3_ESM.zip › Collagen I/CollagenI.doc]

Collagen I-1	GAPDH-1

Collagen I-2	GAPDH-2

Collagen I-3	GAPDH-3
