# Supplementary material for: Human amnion mesenchymal stem cells promote endometrial repair via paracrine, preferentially than transdifferentiation
Source: Cell Commun Signal. 2024 May 31;22:301. doi: 10.1186/s12964-024-01656-0 (PMC11140932; doi:10.1186/s12964-024-01656-0)
Supplement: Supplementary file 4 — Supplementary Material 4: E-cad [file 12964_2024_1656_MOESM4_ESM.zip › E-cad/E-cad.doc]

E-cad-1	GAPDH-1

cad-2	GAPDH-2

E-cad-3	GAPDH-3

35k Da

40k Da

100k Da

150k Da
