# Supplementary material for: Human amnion mesenchymal stem cells promote endometrial repair via paracrine, preferentially than transdifferentiation
Source: Cell Commun Signal. 2024 May 31;22:301. doi: 10.1186/s12964-024-01656-0 (PMC11140932; doi:10.1186/s12964-024-01656-0)
Supplement: Supplementary file 5 — Supplementary Material 5: FN [file 12964_2024_1656_MOESM5_ESM.zip › FN/FN.doc]

FN-1	GAPDH-1

FN-2	GAPDH-2

FN-3	GAPDH-3

40k Da

35k Da

250k Da

150k Da
