# Supplementary material for: Human amnion mesenchymal stem cells promote endometrial repair via paracrine, preferentially than transdifferentiation
Source: Cell Commun Signal. 2024 May 31;22:301. doi: 10.1186/s12964-024-01656-0 (PMC11140932; doi:10.1186/s12964-024-01656-0)
Supplement: Supplementary file 6 — Supplementary Material 6: Vimentin [file 12964_2024_1656_MOESM6_ESM.zip › Vimentin/Vimentin.doc]

Vimentin-1	GAPDH-1

Vimentin-2	GAPDH-2

Vimentin-3	GAPDH-3

40k Da

35k Da

50k Da

70k Da
